# Macrophage cell membrane-based nanoparticles: a new promising biomimetic platform for targeted delivery and treatment

**DOI:** 10.1186/s12951-022-01746-6

**Published:** 2022-12-27

**Authors:** Yuesong Wu, Shengli Wan, Shuo Yang, Haiyang Hu, Chunxiang Zhang, Jia Lai, Jiahan Zhou, Wang Chen, Xiaoqin Tang, Jiesi Luo, Xiaogang Zhou, Lu Yu, Long Wang, Anguo Wu, Qingze Fan, Jianming Wu

**Affiliations:** 1 School of Pharmacy, Southwest Medical University, Luzhou, 646000 Sichuan China; 2 Department of Pharmacy, the Affiliated Hospital of Southwest Medical University, Luzhou, 646000 Sichuan People’s Republic of China; 3 School of Basic Medical Sciences, Southwest Medical University, Luzhou, 646000 Sichuan China; 4 Department of Chinese Materia Medica, School of Pharmacy, Chengdu University of Traditional Chinese Medicine, Chengdu, Sichuan China; 5 Department of Medical Technology, Faculty of Associated Medical Sciences, Chiang Mai University, Chiang Mai, 50200 Thailand

**Keywords:** Biomimetic nanoparticles, Macrophage, Macrophage membranes, Cancer, Inflammation

## Abstract

Synthetic nanoparticles with surface bioconjugation are promising platforms for targeted therapy, but their simple biological functionalization is still a challenging task against the complex intercellular environment. Once synthetic nanoparticles enter the body, they are phagocytosed by immune cells by the immune system. Recently, the cell membrane camouflage strategy has emerged as a novel therapeutic tactic to overcome these issues by utilizing the fundamental properties of natural cells. Macrophage, a type of immune system cells, plays critical roles in various diseases, including cancer, atherosclerosis, rheumatoid arthritis, infection and inflammation, due to the recognition and engulfment function of removing substances and pathogens. Macrophage membranes inherit the surface protein profiles and biointerfacing properties of source cells. Therefore, the macrophage membrane cloaking can protect synthetic nanoparticles from phagocytosis by the immune cells. Meanwhile, the macrophage membrane can make use of the natural correspondence to accurately recognize antigens and target inflamed tissue or tumor sites. In this review, we have summarized the advances in the fabrication, characterization and homing capacity of macrophage membrane cloaking nanoparticles in various diseases, including cancers, immune diseases, cardiovascular diseases, central nervous system diseases, and microbial infections. Although macrophage membrane-camouflaged nanoparticles are currently in the fetal stage of development, there is huge potential and challenge to explore the conversion mode in the clinic.

## Introduction

Most therapeutics, including small molecules and biological macromolecules used in the clinic of various diseases, have to effectively work to act on targets to cells. However, the major problem in the treatment of many diseases is to deliver therapeutics into the target site. These therapeutics are characterized by limited effectiveness, poor biodistribution, and lack of selectivity [1]. Studies have shown that nanoparticle drug delivery systems can overcome these limitations and drawbacks by controlling drug delivery to the inflamed site or tumor so that the influence on normal cells or tissues can be minimized. Currently, an increasing number of nanocarriers have been permitted by the Food and Drug Administration (FDA) on the market, such as poly(D,L-lactic-co-glycolic acid) (PLGA), liposomes, polylactic acid (PLA), polycaprolactone (PCL), iron oxide nanoparticles, nanocrystals and albumin nanoparticles [2, 3].

The transportation of foreign substance nanoparticles to lesions is still a challenge due to the reticuloendothelial system (RES) and the mononuclear phagocytic system (MPS) [4]. When nanoparticles enter the body, they are first bound by proteins that make nanoparticles more recognizable by phagocytic cells. Subsequently, they are rapidly cleared by RES and MPS, which limits their delivery and distribution [5]. For nanoparticles to efficiently enter lesion sites, they need to evade clearance by the immune system. The most common method is to modify the surface of nanoparticles with polyethylene glycol (PEG) [6]. The stability of PEG in vivo, the accelerated blood clearance (ABC) effect and the difficulty of phagocytosis by target cells limit the application [7]. But simple surface functionalization cannot accurately simulate the complex interface in the body and cannot avoid foreign body recognition and subsequent immune responses [8]. To solve this problem, methods based on active targeting have been developed. The surface of the nanocarrier is connected with affinity ligands such as antibodies, peptides, aptamers or other small molecules to bind to target cells through ligand-receptor interactions and release drugs in the target cells [9]. For example, nucleic acid strands as known as aptamers, show relatively strong binding affinity and target specificity, but easily degraded and may cause immune response in body [10]. Protein-based nanomedicine is used in tumor chemotherapy due to their merits in bioavailability, biocompatibility, biodegradability, and low toxicity. Ding et al. have prepared human serum albumin nanoparticles to deliver paclitaxel (NPs-PTX) [11]. It shows that NPs-PTX enhance the cytotoxicity in MCF-7 and A549 cells. Nevertheless, antibodies conjugated nanoparticle encompass high production costs and complex preparation process.

**Fig. 1:**
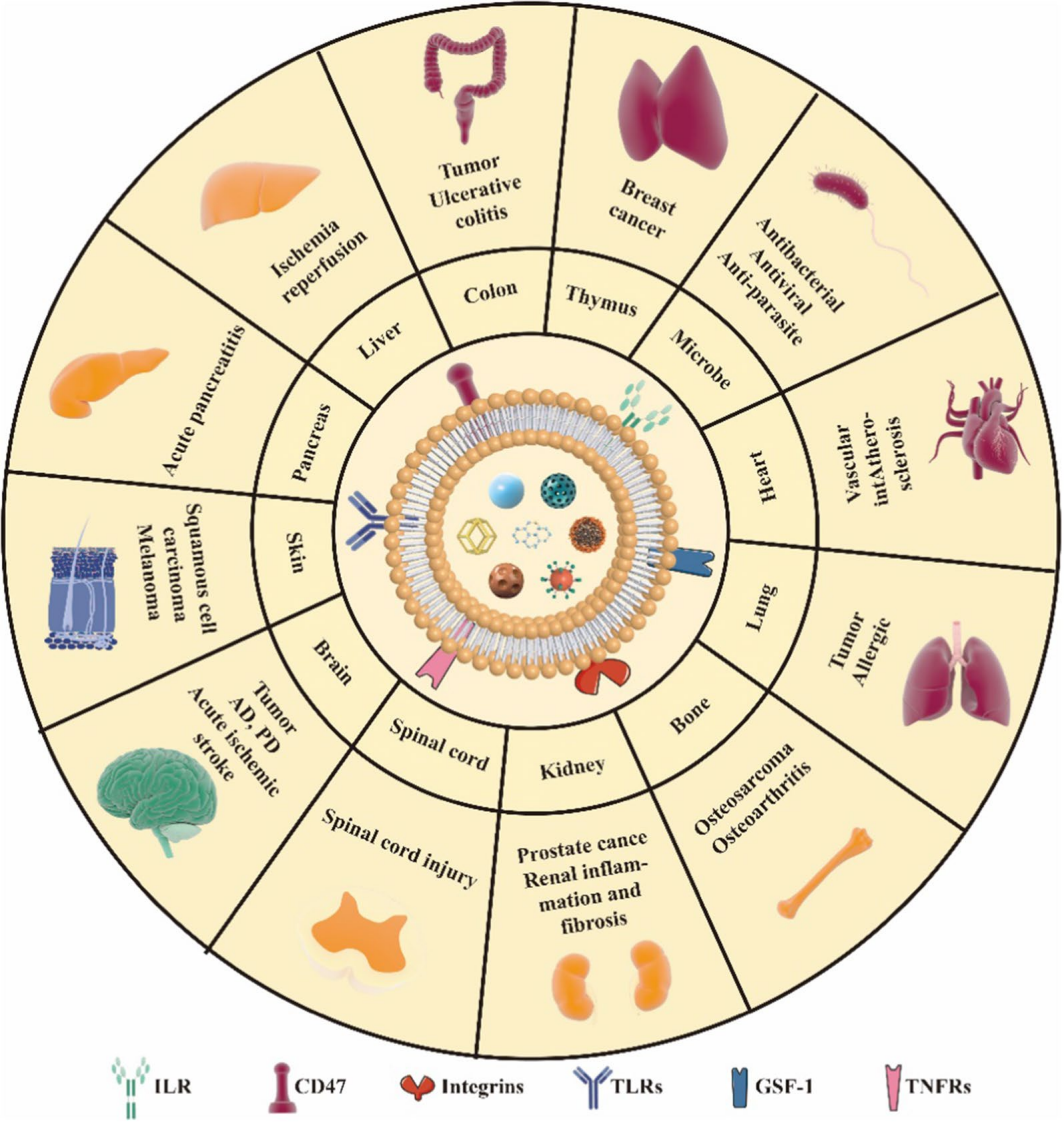
Application of macrophage membrane-coated nanoparticles

Recently, the cell membrane cloaking strategy has emerged as an epidemic means in various diseases. Natural cell membranes have a variety of source cell-relevant functions, such as ‘self’ markers, biological targeting, and correspondence with the immune system [12]. Cell membrane-coated nanoparticles endow them with certain biological functions, including long circulation, targeted recognition, enhanced accumulation at disease sites and deep tumor penetration. To date, this biomimetic strategy has involved various kinds of cells, such as red blood cell membranes [13], macrophage membranes [14], platelet membranes [15], mesenchymal stem cell membranes [16], and cancer cell membranes [17] and so on. Red blood cells were the first cell types used in cell therapy. CD47, a membrane protein widely expressed on the red blood cells, prevents the clearance from the immune system by delivering a “don’t eat me” signal [13]. This approach significantly extends the circulation time of the core nanoparticles, but do not specifically target inflamed site or tumor. Platelet expressing P-selectin can bind specifically to CD44 on the surface of cancer cells, thus platelet membranes coated nanoparticles can target to tumor tissues. Currently, numerous studies focus on the application of platelet membrane biomimetic nanoparticles in various diseases, including cancer, atherosclerosis, and immune diseases [18]. However, it is the difficulty to isolate and culture of platelet that limited its clinical transformation. Similarly, the harsh culture conditions of mesenchymal stem cells increase the cost of fabricating drug delivery systems. Cancer cell membranes can only be applied to tumors [19]. Macrophages are immune cells responsible for protecting the body against infections, clearing foreign invaders, repairing injured tissue, and resisting pathogens. The membranes from macrophages have cellular self-recognition mechanisms to avoid phagocytosis by immune cells, and the ligands inherited from the membrane can bind to receptors at disease sites. Meanwhile, macrophage membrane-coated nanoparticles can recognize and respond to pathogens and immune signals, including bacterial toxins, viruses and inflammatory cytokines. Furthermore, the strategy can activate anticancer immunity. Taken together, macrophage membrane cloaking is a biomimetic platform with promising therapeutic potential. Macrophage membrane cloaking nanoparticles have been applied in tumors, immune diseases, anti-infection, neurological diseases and cardiovascular diseases (Fig. 1).

Here, we summarize the synthesis and characterization of macrophage membrane-coated nanoparticles and their applications in different diseases. Meanwhile, we conclude how the macrophage membrane functions in various diseases. Finally, we put forward some challenges faced at present in their application in the clinic or factory.

## Macrophages and macrophage membranes

The initial response to an infection is mediated by the innate immune system. The process recruits immune cells, including macrophages, dendritic cells, neutrophils, eosinophils, basophils, mast cells, and natural killer cells. Immune cells can use their pattern recognition receptors (PRRs), including Toll-like receptors (TLRs), RIG-I-like receptors (RLRs) and NOD-like receptors (NLRs), to recognize microorganisms through structural patterns, which are expressed only on pathogens called pathogen-associated molecular patterns (PAMPs) [20]. Among the cellular components of the innate immune system, macrophages and neutrophils are the predominant cells responding to pathogens in the body. Regrettably, neutrophils live just 48 h, but macrophages can live several months or more [21]. Macrophages possess the capacity for active targeting, high immune compatibility and long circulation.

Macrophages are phagocytic cells with broad sources, and they are effectors in the process of inflammation and tissue repair. Tissue-resident macrophages can differentiate from circulating monocytes, which develop from hematopoietic stem cells in the bone marrow or during embryonic development in the fetal liver, yolk sac, or dorsal aorta [22]. They are highly specialized cells that mediate tissue homeostasis in all organs, such as splenic macrophages of the spleen, intraocular macrophages of the eye, intestinal macrophages of the gut, osteoclast macrophages of the bone, Kupffer macrophages of the liver, alveolar macrophages of the lungs, Langerhans macrophages of the skin, and microglial cells of the brain [23]. Macrophages are involved in innate immune responses to protect the body by phagocytosing microorganisms and apoptotic cells and presenting antigens [24, 25]. In the early stages of inflammation, macrophages play an important role by releasing cytokines and chemokines that in turn recruit other immune cells to sites of inflammation to start the adaptive response of the immune system.

Macrophages have two phenotypes, named the M1 and M2 phenotypes. M2 is further classified into M2a, M2b, M2c and M2d. Previous studies have shown that lipopolysaccharide (LPS), interferon-γ (IFN-γ) and transforming factor-α (TNF-α) can induce monocytes to produce the M1 phenotype, while interleukin-4 (IL-4), interleukin-10 (IL-10), interleukin-13 (IL-13) transforming factor-β (TNF-β), bacterial infection, colony-stimulating factor 1 (CSF-1) and interleukin-21 (IL-21) are used to produce the M2 phenotype. M1 and M2 macrophages have different functions. M1 macrophages mainly play a role through innate and adaptive immune responses in Th1-cell recruitment, pathogen resistance, and tumor control [26]. They produce proinflammatory factors, including IL-1, IL-6, TNF-α, NO and reactive oxygen species (ROS), to promote T cells to produce Th1 cytokines [27]. Therefore, under certain conditions, M1 macrophages exacerbate inflammatory processes, which are detrimental to healthy tissue. In contrast to M1 macrophages, M2 macrophages express opposite functions, responding to parasites, tissue remodeling, angiogenesis, and allergic diseases by downregulating IL-12 and IL-23 but upregulating IL-10 and IL-1RA [28]. The M2 type promotes the release of anti-inflammatory cytokines, wound healing, and the growth and metastasis of tumor cells in the tumor microenvironment [29, 30]. In summary, M1 macrophages kill pathogens by recruiting inflammatory cells in the process of tissue inflammation. M2 macrophages are used to inhibit the recruitment of inflammatory cells to protect against excess proinflammatory cytokines and promote angiogenesis and tissue repair. However, how did this process develop?

Studies have shown that there is a special recognition mechanism between macrophages and pathogens. This process is primarily initiated by PAMPs and damage-associated molecular patterns (DAMPs) in response to infection and injury [31, 32]. The migration of macrophages depends on the expression of adhesive molecules by chemical mediators on venous endothelial surfaces. Cell adhesion molecules are cell surface proteins that mediate cell-cell and/or cell-extracellular matrix (ECM) interactions. These adhesion molecules contain integrins, selectins, cadherins, immunoglobulin superfamily and others. Macrophage membranes express P-selectin glycoprotein ligand-1 (PSGL-1), L-selectin, lymphocyte function-associated antigen 1 (LFA-1), integrin, and very late antigen-4 (VLA-4), which result in their cell adhesion with inflammatory cells and cancer cells. It has been suggested that macrophage membrane-cloaking nanoparticles can actively target tumors by binding with integrin to vascular cell adhesion protein 1 (VCAM-1) or LFA-1, which is highly expressed in active cell membranes [33–36].

Therefore, using these properties of macrophages, macrophage membrane cloaking nanoparticles can avoid clearance by the immune system and achieve tumor as well as inflammatory tissue targeting capacity. This strategy has shown great potential to overcome the biocompatibility, short cycle time, and immunogenicity of traditional materials.

## Fabrication of macrophage membrane biomimetic nanoparticles

### Multistep synthesis of cell membrane cloaking nanoparticles

**Fig. 2:**
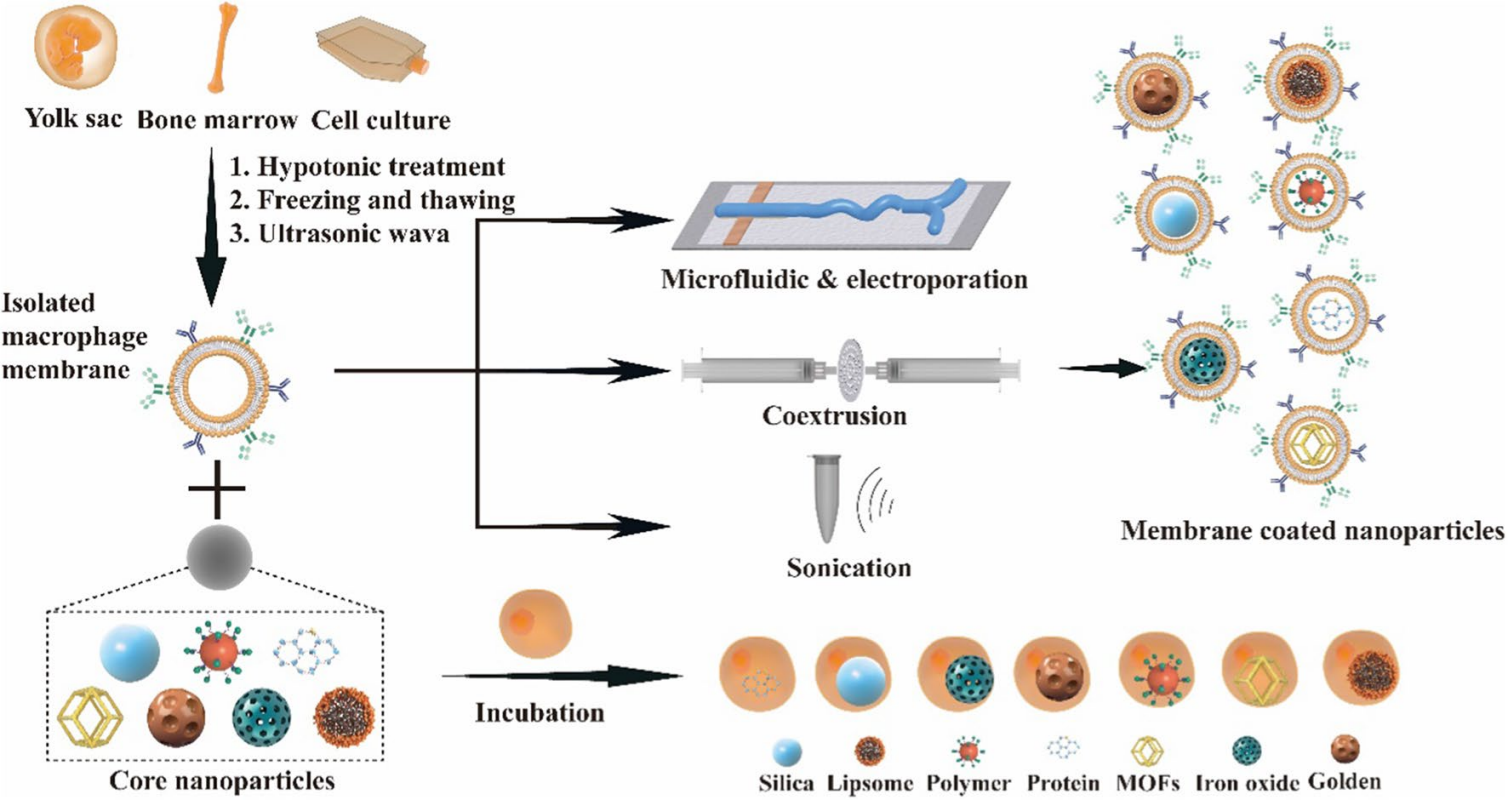
The development of macrophage membrane-coated nanoparticles with different methods

Cell membrane-camouflaged nanoparticles are usually composed of a thin layer of cell membrane encapsulating therapeutic nanoparticles, thus forming a “shell-core” structure with nanoparticles as the core and the cell membrane as the outer shell [37]. The synthesis of macrophage membrane-coated nanoparticles usually involves the following three steps [38–40]: (1) macrophage cell membrane extraction, (2) fabrication of the core, and (3) the formation of membrane-wrapped nanoparticles (Fig. 2).

#### Source and isolation of macrophage membrane

Previous studies have shown two major classes of macrophage membranes, primary macrophages isolated from animals and macrophages cultured in plates. Cells are composed of membranes, intracellular biomacromolecules, intracellular vesicles, and nuclei. Membrane extraction needs to remove these intracellular components while leaving the whole functional surface protein of membranes. These proteins can transport molecules in or out of the cells, correspond between the cell and cell and are related to other processes [41, 42]. Proteins on the cell membrane are important for biomimetic therapy because they can endow bioinspired nanoparticles with targeting ability and immune escape capacity. Therefore, the isolation of the cell membrane needs to be carried out moderately to reduce the denaturation of membrane proteins [43]. Usually, this process requires a mass of cells to be harvested from culture dishes such as RAW264.7, J774A.1, THP-1 and blood or tissues [44, 45]. The harvested cells can be subjected to hypotonic treatment, freeze-thaw cycling and ultrasonic waves. After discontinuous sucrose gradient centrifugation, the pellets were collected. Then, the cell suspension was disrupted by ultrasound with appropriate power and extrusion through a porous polycarbonate membrane to obtain the membrane vesicles required for the experiment. Extracted macrophage membranes are stored together with protease inhibitors at 4 °C to maintain the stability of biological activity [46].

#### Inner core

The selection of the inner core depends on the intended application, as they are the effective loads delivered to targeted tissues [47]. They are particulate dispersions or solid particles with a particle size in the range of 10–1000 nm [48]. In recent years, various types of materials for cell membrane cloaking have been widely explored and applied, including PLGA, liposomes, SiO_2_, mesoporous silica, nanocapsules, gold, iron oxide, upconversion nanoparticles (UCNPs) and metal-organic frameworks (MOFs) (Table 1). The inner cores can be classified as organic and inorganic nanoparticles in terms of the properties of materials [49]. Organic particles are formed by organic molecules through a self-assembly process, including polymeric nanoparticles and lipid nanoparticles [50]. They have simple design principles, high biocompatibility and high drug-loading capacity. However, the high cost of organic materials and manufacturing equipment, poor stability in vivo, difficulty in controlling size, large differences between different batches and residual organic solvents limit the application of organic cores [50]. Inorganic cores include silica nanoparticles [14], gold nanocage (AuNC) nanoparticles [51], iron oxide nanoparticles [52], upconversion nanoparticles (UCNPs) [53], metal-organic frameworks (MOFs) [54]. They have various advantages in terms of controllable size, good stability, large specific surface area for surface functionalization. Meanwhile, their unique photothermal or electromagnetic properties give them potential therapeutic and imaging functions. However, the low biodegradability and high toxicity in vivo of inorganic vectors hinder their clinical transformation [55].

**Table 1:** Types of core nanoparticles coated by macrophage membrane

Materials	Size (nm)	Zeta potential (mV)	References
PLGA	85.8	− 42.4	[102]
Gold nanocage	~ 100	~ − 30	[162]
MOFs	37.8	23.3	[54]
Liposome	64.5	− 28.0	[35]
Bismuth selenide	145.6	− 27.8	[146]
MSNs	47.8	− 7.5	[14]
UCNPs	88.6	− 35	[147]
Human serum albumin	138.7	− 15.7	[80]
Chitosan	237.5	− 0.7	[148]
Fe_3_O_4_	80	/	[38]
CuS	290	− 10.6	[88]
Polydopamine	90	3.4	[66]
Solid lipid	109	− 25	[127]

#### Preparation of macrophage membrane biomimetic nanoparticles

The last and most important step is to coat the prepared macrophage membrane on the synthetic nanoparticles. Currently, there are four methods to prepare macrophage membrane biomimetic nanoparticles: incubation, membrane extrusion, sonication or electroporation. An incubation approach has been described in which nanoparticles with an indicated concentration are incubated with cells for several hours [56]. This method is good for maintaining the stability of membrane proteins. However, few macrophage-coated nanoparticles are collected. For membrane extrusion, cell membrane vesicles and nanoparticles are coextruded several times through polycarbonate porous membranes of different pore sizes, such as 200 nm, to develop the biomimetic nanoparticles. In physical extrusion, the mechanical force prompts them to form “shell-core” structures by disrupting the membranes and reforming them [15, 57–60]. Although this strategy does not require sophisticated equipment, large-scale production and integrity of membrane proteins have become major obstacles to clinical application. In sonication-based methods, the cores are coincubated with cell membrane vesicles and ultrasound at an appropriate parameter; this process is easy to achieve in the clinic or factory and prepares good shape and size cell membrane-coated nanoparticles [61, 62]. Recently, electroporation has become a new approach to prepare macrophage-coated nanoparticles. Cores and cell membrane vesicles are infused into a microfluidic chip, and then, electric pulses produce transient pores in the membranes to promote NPs into membrane vesicles. This strategy shows excellent advantages in maintaining the integrity of the nanovesicles and a higher success of the CM-NPs [63].

### Characterization of macrophage membrane coated nanoparticles

**Fig. 3:**
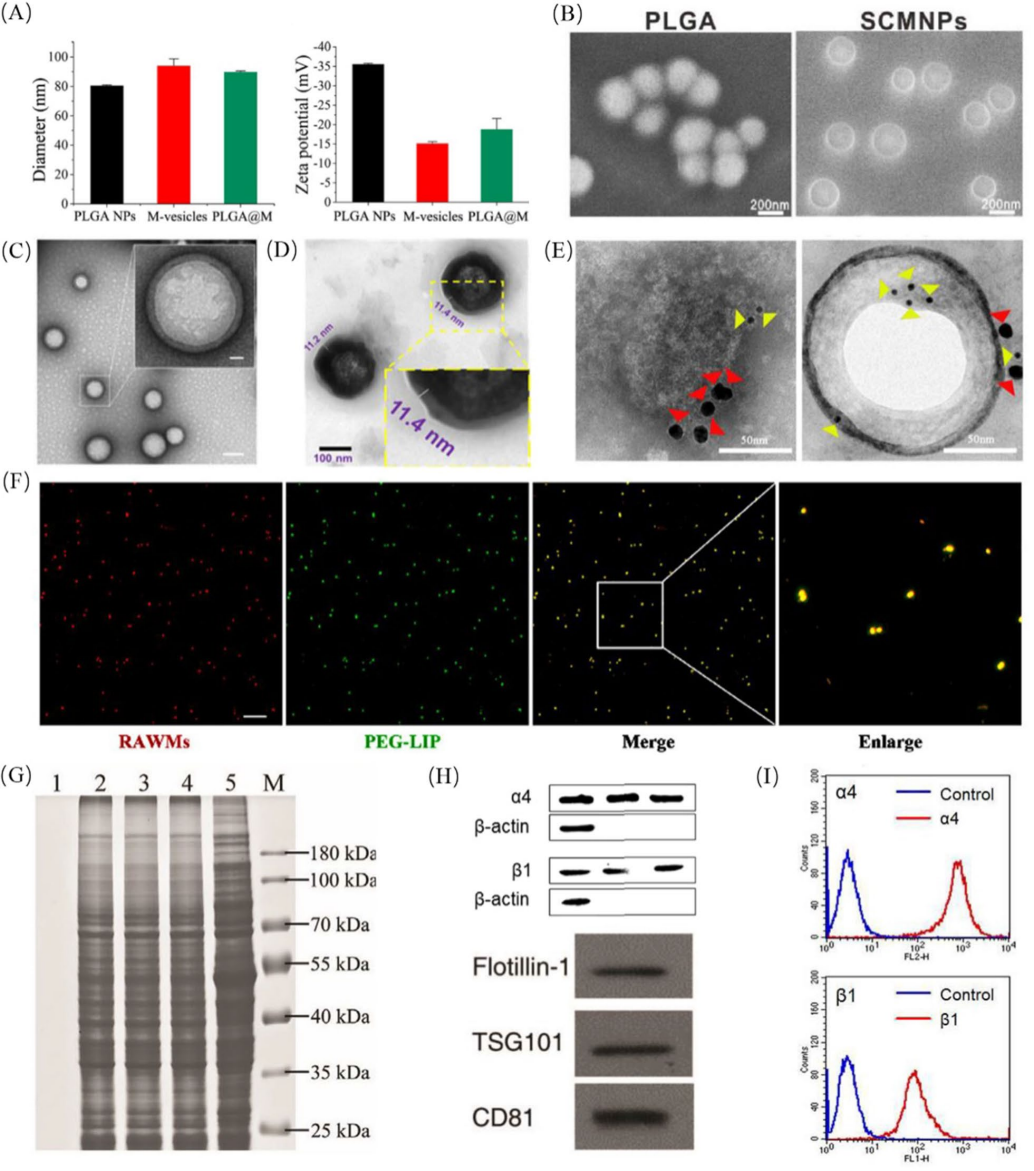
Characterization of cell-membrane coated nanoparticles. **A** Hydrodynamic diameter and zeta potential of PLGA NPs, M-vesicles and PLGA@M after formulation in water. Adapted with permission from [102], copyright ^©^ 2022 BioMed Central Ltd unless otherwise stated. Typical SEM (B) and TEM (C, D) image of core nanoparticles before and after cell-membrane coating. **B** Adapted with permission from [68], Copyright ^©^ 2022 Elsevier B.V. **C** Adapted with permission from [94], Copyright ^©^ 2022 National Academy of Science. **D** Adapted with permission from [33], copyright ^©^ 2020 American Chemical Society. **E** Immunogold TEM images of membrane and membrane coated NPs samples probed for α4 (red arrows) and vascular cell adhesion molecule-1 (yellow arrows). Adapted with permission from [72], copyright ^©^ 2022 BioMed Central Ltd unless otherwise stated. **F** Co-localization of membranes (red) and core (green) by CLSM. Adapted with permission from [131], copyright ^©^ 2022 Elsevier B.V. **G** Representative SDS-PAGE result showing the membrane proteins analysis of PLGA cores (lane 1), AM membranes (lane 2), AM vesicles (lane 3), TN@AM NPs (lane 4), and AM cell lysate (lane 5). Lane M: marker. Adapted with permission from [36], ^©^ 2021 The Authors. Advanced Science. **H** Western blotting analysis demonstrating the retention of characteristic membrane proteins. Adapted with permission from [33], copyright ^©^ 1999–2022 John Wiley & Sons, Inc. **I** The α4 and β1 integrins in RAW264.7 cells measured by FACS analysis. Adapted with permission from [35], copyright ^©^ 2016 American Chemical Society

The macrophage membrane-coated nanoparticles were further investigated to confirm that the macrophage membrane successfully coated the surface of the NPs. Physicochemical and biological properties, such as size, zeta potential, and membrane protein composition, need to be characterized, as does the function of macrophage-coated nanoparticles (Fig. 3).

#### Physicochemical properties

Successful wrapping can be validated by observing a 10–20 nm increase in particle size, which is equal to the thickness of the membrane layer, and the charge of membrane-coated nanoparticles should be close to the potential of the macrophage membranes [64]. To prepare membrane-coated nanoparticles, Wang et al*.* designed the optimal ratio of microvesicles at different mass ratios to screen uniform and stable sizes and zeta potentials by dynamic light scattering (DLS) [65]. DLS is one of the most accurate and economical methods to determine the size distribution and zeta potential of nanoparticles. Wei et al*.* reported that the mean size of membrane-coated nanoparticles increased by 10–15 nm compared with that of bare nanoparticles, and the zeta potential of membrane-coated nanoparticles was close to that of macrophage membranes (Fig. 3A) [66]. The naked nanoparticles can form a distinct “shell-core” structure after coating by macrophage membranes. Scanning electron microscopy (SEM) and transmission electron microscopy (TEM) are mainly used to examine the structure and morphology of the sample, such as size, shape, aggregation and surface morphology. SEM images revealed that the bare core and macrophage-coated core exhibited a spherical shape with a smooth surface (Fig. 3B). TEM images showed a typical ‘core–shell’ structure and a single dimer macrophage membrane layer (~ 10 nm) on the core nanoparticles (Fig. 3C, D). Recently, immunogold staining fluorescence and colocalization analysis have been used to verify the success of the membrane coating through a method of visual evidence. Immunogold staining showed that the specific markers were simultaneously present on the surface of the core (Fig. 3E). Colocalization assays showed the overlap of fluorescence of macrophage membranes and core, indicating the success of membrane coating (Fig. 3F). Infrared spectroscopy, nuclear magnetic resonance spectroscopy and mass spectrometry are also required to determine the successful preparation of drugs or nanoparticles. Finally, in vitro release experiments are also required to measure the release rate of the drug under different acidic environments.

#### Biological function properties

Particle size, zeta potential, electron microscopy and others confirmed the success of the membrane coating. However, the integrity of the membrane components and the retention of membrane protein activity directly determine whether the encapsulated nanoparticles can perform their specific functions. Therefore, to detect the retention of membrane activity, we can examine its protein profile as well as the concentration of characteristic proteins. Western blotting and sodium dodecyl polyacrylamide gel electrophoresis (SDS-PAGE) were used to analyze the protein environment of the biomimetic nanoparticles. SDS-PAGE was used to verify the successful migration of overall proteins on the cell membrane and compare the difference in membrane proteins present on the cell membrane of source cells, extracted membrane and cell membrane-camouflaged nanoparticles (Fig. 3G). Furthermore, western blotting was validated against key membrane proteins with specific protein markers (Fig. 3H). As shown in Table 2, integrin α4 and integrin β1 are usually detected in macrophage membranes because they can actively bind to vascular cell adhesion molecule-1 of cancer cells or inflamed endothelium. CD63 and TSG101 are usually detected in M-exo and macrophage-derived microvesicles. In addition, flow cytometry and qPCR can also verify membrane proteins and mRNAs. For example, Rao et al. made use of flow cytometry and qPCR to verify the success of genetically modified cell membranes (Fig. 3I).

**Table 2:** Macrophage and macrophage-derived exosome membrane surface markers that may contribute to nanomaterials’ application

Membrane source	Specific marker	Ligands	Function	Refs.
Macrophage membrane	Integrin α4β1	VCAM-1	Cell adhesion molecule: actively bind to VCAM-1 on cancer cell	[33, 35, 40, 53, 66, 110, 111, 114, 115, 132, 149, 155, 165, 168]
	MAC-1	VCAM-1	Bind to flamed HUVECs or tumor tissue	[132, 139, 168]
	CD47	SIRP-α receptor	Prevent the undesirable phagocytosis by inhibiting binding to receptor SIRP-α pathway	[111, 114, 115, 165]
	CCR2	CCL2	Promote the recruitment of monocytes to “home” the inflammatory lesion	[112, 165]
	CD36	Receptor for oxLDL	Target oxLDL	[112]
	TNFR2	TNF	Adsorb cytokines	[112, 162]
	CD66a	Spike protein receptor	Target and bind to the Spike protein of coronavirus	[36, 102]
	CD126	IL-6 receptor	Adsorb cytokines	[36, 94, 102, 162]
	CD119	IFN-γ receptor	Adsorb cytokines	[36, 94, 102]
	CD135	Tyrosine kinase 3 receptor	Directly bind extracellular ligands and transduce regulatory signals	[68, 88]
	CD309	VEGF receptor	Bind to VEGF and induce cell proliferation and migration	[68]
	CD44	Extracellular matrix components and messenger molecules	Promote tumor progression and metastasis	[68, 139]
	CSF1R	CSF1	Activates the downstream signaling pathway responsible for the polarization of TAMs to the immunosuppressive phenotype	[75]
	TLR2/4	LPS receptor	Recognize LPS of Gram-negative bacteria and activates NF-κB	[94, 97, 166]
	F4/80	EGF receptor	Macrophage markers in mature mice and cell-to-cell interactions	[75, 127, 140, 166]
	CD206	Mannose receptors	M2 marker	[75, 116, 140, 166]
	CD11a/b/c	ICAMs	Mediate cell adhesion	[75, 84, 127, 166]
	CD14	LPS receptor	Recognize LPS of Gram-negative bacteria and activates NF-κB	[94]
	CD130	IL6/IL6R complex	Signal-transducing molecule	[94]
	CD120a/b	TNF-α/β	Adsorb cytokines and activate macrophage	[94]
	CD68	-	Macrophage marker	[140]
Exosomes derived from macrophages	Arginase-1	Arginine	M2 marker	[137]
	CD163	Bacterial; LPS	M2 marker; participation in immunization	[116, 137]
	CD9	-	Anti-inflammation marker; cell adhesion; cell motility; tumor metastasis	[65, 150, 173]
	LAMP2	Lectin -1/3 receptor	Cell adhesion; tumor metastasis	[173]
	ALIX	-	Involved in exosome biogenesis	[71, 173]
	TSG101	-	Exosome marker	[65, 71, 84, 131, 136, 171, 173]
	CD63	-	Exosome specific marker	[71, 116, 131, 136, 150]
	Calnexin	Lectin receptor	Endoplasmic reticulum marker	[136]
	CD81	-	Exosome marker	[65, 150, 171]
	Flotillin-1	-	Involved in exosome biogenesis	[84, 171]

## Application of macrophage membrane biomimetic nanoparticles

Macrophage membranes inherit the function of the natural membranes so that endow the nanomaterials  with immune evasion, enhanced compatibility, ability to target inflammation and tumors. Recently, this biomimetic strategy has been employed successfully in the treatment of various of diseases including cancers, infection diseases, cardiovascular diseases, CNS diseases, immune disease, and other inflammatory. The macrophage membranes coated nanoparticles have a capacity to target tumors and inflamed tissues by the surface receptors such as integrins, MAC-1 and CSF1R. Table 3 has summarized the biomedical applications of macrophage membranes coated nanosystems.

**Table 3:** The application of macrophage-coated nanoparticles

Disease		Membranes source	Core	Cargos	Cell lines	Characters	Refs.
Cancers	Breast	RAW264.7	CuS	PTX	In vitro: 4T1 In vivo: 4T1	1. Dual anticancer action (PTT) and chemo) 2. Deep tumor penetration 3. Excellent tumor accumulation and retention	[33]
		BMMs	-	-	In vitro: 4T1 In vivo: 4T1 and B16F10	1. Programmable cellular vesicle 2. Recognize and interact with CTCs 3. Cancer immunotherapy	[81]
		RAW264.7	PLGA	siRNA	In vitro: 4T1 In vivo: 4T1	1. Combine immune-metabolic and immune checkpoint inhibitors 2. pH-responsive platform 3. Improve the endo-lysosomal escape 4. Hybrid biomimetic membrane	[72]
		RAW264.7	Gold	DOX	In vitro: 4T1 In vivo: 4T1 and B16F10	1. M1 macrophage membrane 2. Size-changeable 3. Prolong circulation retention properties 4. Cancer immunotherapy	[145]
		RAW264.7	PLGA	DOX	In vitro: 4T1 In vivo: 4T1	1. Hybrid membrane 2. Accumulate at sites of inflammation 3. Target specific metastasis 4. Homogenous tumor targeting abilities	[40]
		RAW264.7	PLGA	Saikosaponin D	In vitro: 4T1 and MCF-7 In vivo: 4T1	1. Macrophage membrane hybridized with T7 peptide 2. Anti-angiogenesis	[68]
		RAW264.7	Liposome	Emtansine	In vitro: 4T1 In vivo: 4T1	1. Improve specific metastasis targeting capability 2. Enhance cellular uptake by 4T1 cells	[35]
		RAW264.7	Liposome	DOX	In vitro: 4T1 In vivo: 4T1	1. Bio-imagining 2. Anti-metastasis 3. Evade the immune system’s response	[53]
		Mouse peritoneal macrophage	-	PTX	In vitro: MDA-MB-231 In vivo: orthotopic tumor model	1. Tumor homing 2. Step-by-step controlled drug release 3. Cascade-responsive polymeric nanoparticles	[67]
		RAW264.7	Bi_2_Se_3_	Quercetin	In vitro: 4T1 In vivo: 4T1	1. Prolong circulation life 2. Enhance tumor-itropic accumulation 3. Minimal side effect 4. PTT and chemo	[146]
		RAW264.7	Mesoporous silica	DOX	In vitro: NIH/3T3 In vivo: 4T1	1. Good stability in vivo 2. Reduce retention RES organs and prolong blood-circulating time 3. Effective accumulation in tumors	[14]
		RAW264.7	UCNPs	-	In vitro: MCF-7 In vivo: MCF-7	1. Effective cancer targeting 2. Cancer imaging 3. Good in vivo biocompatibility	[147]
		THP-1	Chitosan	-	In vitro: hela, MDA-MB-231 and MCF-7 In vivo: 3D tumor spheroids of Hela	1. Membrane-bound TNF-α 2. Excellent biocompatibility 3. Innate anti-cell proliferative	[148]
		Tumor-associated macrophage	UCNPs	Rose Bengal	In vitro: MCF-7 and 4T1 In vivo: 4T1	1. Enhanced biocompatibility and immune compatibility 2. Competitive inhibition 3. A promising immunotherapeutic agent	[75]
		RAW264.7	Liposome	DOX	In vitro: 4T1 In vivo: 4T1-luc cells	1. Maximally reduce side-effects 2. pH-sensitive prodrug 3. Enhance therapeutic efficacy	[149]
		RAW264.7 derived exosome	AA-PEG	PTX	In vitro:3LL-M27 In vivo: 3LL-M27	1. High loading capacity 2. Exosome modified with AA-PEG	[84]
		J774A.1 derived exosome	Liposome	DOX	In vitro: 4T1, K7M2, NIH/3T3 In vivo: -	1. High colloidal stability 2. pH-sensitive sustained drug release 3. Tumor-targeted drug delivery	[150]
		RAW264.7	-	DOX	In vitro: 4T1 In vivo: 4T1	1. Definite tumor homing ability 2. Macrophage incubate with DOX solution 3. Treat lung metastasis	[151]
		RAW264.7 derived exosome	Liposome	DOX	In vitro: T11 and MDA-MB-231 In vivo: T11 and MDA-MB-231	1. High drug loading 2. Efficient accumulation in triple negative breast cancer cells 3. High anticancer efficacy	[152]
		C57Bl6 IC21 macrophages or Balb/c RAW264.7 macrophages	Lipoplex	siRNA	In vitro: 4T1, MDA-MB-231 and 3LL In vivo: MDA-MB-468	1. Deliver nucleic acids into solid tumor 2. Accumulate at breast cancer 3. Incubation method	[153]
		M1 macrophage derived exosome	-	PTX	In vitro: 4T1 In vivo:4T1	1. Immunological-chemo therapy 2. M1 macrophage exosome 3. High antitumor effects	[71]
		RAW264.7	Liposome and Gold nanorods	DOX	In vitro: 4T1 In vivo: 4T1	1. PTT and chemotherapy 2. Enhance tumor penetration and coverage 3. Enhance the therapeutic therapy	[154]
		RAW264.7	FeO	Carbonic anhydrase IX	In vitro: 4T1 In vivo: 4T1	1. Chemo-dynamic therapy 2. Tumor microenvironment remodeling 3. Prolong the blood circulation time	[155]
		RAW264.7	Prussian blue	Hydroxychloroquine	In vitro: 4T1 In vivo: 4T1	1. Macrophage repolarization 2. Reduce RES uptake 3. Enhance tumor accumulation	[156]
		RAW264.7	Gold and silica	-	In vitro: 4T1 In vivo: 4T1	1. PTT 2. Long circulation 3. Good colloidal stability	[62]
		Bone marrow derived macrophages	PLGA	UCNPs	In vitro: NIH/3T3 and 4T1	1. Bio-imaging 2. Deep penetration 3. NIR-response	[74]
		RAW264.7	Chlorin e6	PTX	In vitro: 4T1 In vivo: 4T1	1. Shape changeable nanomedicine 2. Sustain release drugs 3. Induce immunogenic cell death 4. PDT	[157]
	Skin	J774A.1	MOF-derived mesoporous carbon	Doxycycline hydrochloride, acetylsalicylic acid, PTX	In vitro: SCC-7 In vivo: SCC-7	1. Enhanced drug loading capacity 2. Evade the mononuclear phagocyte system 3. Autofluorescence-free persistent luminescence imaging	[158]
		RAW264.7	Albumin	PTX	In vitro: B16F10 In vivo: B16F10	1. Enhanced cytotoxicity and apoptosis rates 2. Prolong blood circulation 3. Selective accumulation at tumor site	[54]
		J774.1 THP-1	Nano-porous silicon	DOX	In vitro: MDA-MB-231 In vivo: MDA-MB-231	1. Enhanced circulation time 2. Improved accumulation in tumor 3. Evade the immune system 4. Cross the biological barriers	[80]
	Prostate	RAW264.7	Graphene	DOX	In vitro: RM-1 In vivo: RM-1	1. Effective combination of chemotherapy and PTT 2. Active targeting of tumor cells 3. Stimuli-releasee triggered by NIR	[79]
	Cervix	THP-1	SiO_2_	-	In vitro: Hela	1. PTT 2. Enhance tumor penetration 3. Achieve good biocompatibility	[87]
	Lung	J774A.1	-	DOX	In vitro: HUVEC In vivo: LL/2	1. Favorable accumulation properties into the lungs 2. No observable acute side effects 3. Simple fabrication protocol	[82]
		mouse peritoneal macrophage	Liposome	DOX	In vitro: A549 In vivo: A549	1. Time-controlled release of drug 2. Targeted tumor site 3. Effective therapeutic efficacy 4. Imaging capacity	[85]
	Liver	RAW264.7	CuS	sorafenib	In vitro: HepG2 In vivo: H22	1. Selectively accumulation both in vitro and in vivo 2. Combination of PTT and chemotherapy 3. Inhibit tumor cell proliferation and angiogenesis	[88]
	Osteosarcoma	RAW264.7	PLGA	PTX	In vitro:143B In vivo:143B	1. Inflammatory chemotaxis 2. Reduce immune clearance 3. Targeted chemotherapy of osteosarcoma	[159]
Anti-microorganism	Antibacterial	J774	PLGA	-	In vitro: murine TLR4 reporter cells In vivo: escherichia coli	1. Bind and neutralize endotoxins 2. Sequester proinflammatory cytokines	[94]
	Antiparasite	RAW264.7	-	Amphotericin B	In vitro: infected macrophage by *L. donovani*	1. Deliver drug to the infected tissues 2. Improved the toxicity profile 3. Lowered LD50 value	[105]
	Antibacterial	RAW264.7	Gold-silica	-	In vitro: *S. aureus* In vivo: *S. aureus*	1. Targeted bacteria more efficiently 2. Prolonged blood circulation time 3. Excellent biocompatibility 4. Combine with PTT	[97]
	Antibacterial	J774 derived exosomes	Liposome	-	In vitro: mouse pulmonary vein endothelial cells In vivo: LPS-induced sepsis model	1. No effect with endothelial cells 2. Decrease proinflammatory genes but anti-inflammatory 3. Regulate the inflammatory response in target cells	[95]
	Antibacterial	J774	PLGA	-	In vitro: J774 In vivo: *P. aeruginosa*	1. Clear pathogens 2. Natural affinity for virulence factors 3. Neutralize both hemolytic and cytotoxic activities 4. Inherently multi-antigenic and safe for in vivo administration	[96]
	Antibacterial	RAW264.7	Ca_3_(PO_4_)_2_-Fe_3_O_4_	-	In vitro: S. aureus; Escherichia coli; MRSA In vivo: MRSA	1. Reserved the integrality of membrane structure 2. Superior properties of recognition and adsorption with bacteria, toxins and inflammatory cytokines 3. Better antibacterial and anti-inflammatory abilities 4. Enhance the tissue repair process	[160]
	Antiviral	THP-1 or BMMs	-	-	In vitro: hepatitis C from patients In vivo: hepatitis C from patients	1. Long-lasting inhibitory effects on HCV 2. High uptake of omega-6pufas 3. Sufficient adaptive immune responses	[161]
	Antiviral	Alveolar macrophages	PLGA	-	In vitro: In vivo: murine hepatitis virus A-59	1. Block coronavirus from host cell entry 2. Absorb proinflammatory cytokines 3. Combine with PTT to disrupt virus 4. Alleviate infection progression and reduce transmission risk	[36]
	Antiviral	THP-1	PLGA	Lopinavir	In vitro: virus infected A549 model In vivo: mouse hepatitis virus	1. Neutralize multiple proinflammatory cytokines 2. Suppress the activation of macrophages and neutrophils 3. Significant targeted ability to inflammation sites 4. Superior therapeutic efficacy	[102]
	Antiviral	Human monocyte-derived macrophage	Liposome	Indinavir, ritonavir, atazanavir, efavirenz	In vitro: monocyte-derived macrophage with HIV-1	1. Prolonged plasma drug concentrations 2. Slow and steady drug release 3. Targeted delivery to infected sites 4. Reduced toxicity	[51]
	Antiviral	BMMs	Liposome	Indinavir	In vitro: HIV In vivo: HIV-infected humanized immune-deficient mice	1. Promote sustained “local” drug release more than 2 weeks 2. Robust lung, liver, and spleen distribution 3. Induce CD^4 +^ T-cell protection	[56]
	Antibacterial	RAW264.7	Gold	Resolvin D1	In vitro: RAW264.7 In vivo: femoral defect model	1. LPS-pretreated membrane 2. Inhibit proinflammation and promote antiinflammation 3. M2 macrophage repolarization	[162]
	Antiviral	BMMs	Liposome	Indinavir	In vitro: HIV-1 infected macrophage In vivo: HIV-1 encephalitis rodent model	1. Readily penetrate the BBB and enhance brain drug distribution 2. Target to disease sites of viral replication and neuroinflammation 3. Improved antiviral efficacy	[101]
	Antibacterial	Peritoneal macrophages	Polymer	-	In vitro: peritoneal macrophages In vivo: sepsis model	1. Reduce the pro-inflammatory cytokines levels 2. Suppress nitric acid, iNOS, and COX-2 3. Increase the medical product for patients who affected sepsis	[163]
Cardiovascular Diseases	Atherosclerosis	THP-1	-	Catalase	In vitro: HUVEC In vivo: -	1. Specificity targeted to damaged endothelial cells 2. Easily fabricated in biological conditions and keep antioxidants active 3. Consume ROS	[164]
		RAW264.7 derived M2 exosomes	-	hexyl 5-aminolevulinate hydrochloride	In vitro: HUVEC In vivo: acute peritonitis mouse model	1. AS imaging ang therapy 2. Molecularly engineered M2 macrophage-derived exosome	[116]
		RAW264.7 derived exosomes	Polymer	Rapamycin	In vitro: HUVEC, RAW264.7 and smooth muscle cells In vivo: embryos from zebrafish	1. Favorable hydrodynamic size with negative surface charge 2. ROS-responsive drug release 3. Effectively escape from macrophage uptake 4. Targeted to inflammatory endothelial cells 5. Nontoxic both in vitro and in vivo	[114]
		RAW264.7	Polymer	Atorvastatin	In vitro: HUVEC and RAW264.7 In vivo: ApoE^−/−^ mice	1. Avoid the clearance from the reticuloendothelial system 2. Lead NPs to the inflammatory tissues 3. ROS-responsiveness of NPs enables payload release 4. Sequesters proinflammatory cytokines to suppress local inflammation	[112]
		RAW264.7	Fe_3_O_4_	-	In vitro: H9C2 cells In vivo: atherosclerosis model	1. High biosafety 2. Effectively target early atherosclerotic lesions 3. Bioimaging by MRI	[110]
		RAW264.7	PLGA	Rapamycin	In vitro: HUVEC, smooth muscle cells and RAW264.7 In vivo: ApoE^−/−^ mice	1. Good biocompatibility 2. Inhibit the phagocytosis by macrophages 3. Targeted endothelial cells and accumulated in as lesions 4. Favorable safety performance	[111]
		RAW264.7	PLGA	Colchicine	In vitro: HUVEC and RAW264.7 In vivo: vulnerable atherosclerotic plaque mice	1. Target endothelial cells and escape the endocytosis of macrophage 2. Reduce the lipid plaque load and improve the plaque stability	[115]
	Vascular intimal hyperplasia	RAW264.7	Polymer	Rapamycin	In vitro: vascular smooth muscle cells In vivo: carotid artery wire injury mouse model	1. Innate “homing” capacity 2. Avoid clearance by the immune system 3. Effectively the solubility of rapamycin 4. ROS accumulated for controlling local cargo release 5. Reduce toxic side effects	[165]
	Myocardial ischemia/reperfusion injury (MI/RI)	RAW264.7	Polydopamine	-	In vitro: hypoxia/reoxygenation (H/R) model primary neonatal rat cardiomyocytes In vivo: MI/RI model	1. Target the infarcted myocardium 2. Effectively relieved the MI/RI-induced oxidative stress 3. Inhibit cell pyroptosis by suppressing the NLRP3/caspase-1 pathway	[66]
	Hepatic ischemia-reperfusion injury	RAW264.7	PLGA	-	In vitro: RAW264.7 In vivo: rat liver transplantation model	1. Neutralized endotoxin and suppressed the secretion of inflammatory factors 2. Effectively alleviate MI/RI induced by liver transplantation	[166]
CNS	PD	RAW264.7 derived exosome	PLGA	Catalase	In vitro: PC12 In vivo: mouse model of PD	1. High loading efficiency 2. Sustained release 3. Readily taken up by neuronal cells 4. Significant neuroprotective effects in in vitro and in vivo	[125]
	PD	BMMs	Polymer	Catalase	In vitro: mouse catecholaminergic CATH.a neurons	1. Targeted therapeutic tissue-specific delivery 2. Sustained release of catalase and to enter the brain 3. Protect nigrostratial neurons 4. Increase BBB penetration and ROS decomposition	[126]
	PD	BMMs	Polymer	Catalase	In vitro: - In vivo: mouse model of PD	1. Attenuate neuro inflammation and bigrostriatal degeneration 2. Prolong blood circulation time 3. Targeted diseased sites 4. Bioimaging	[123]
	PD	BMMs	Polymer	Catalase	In vitro: - In vivo: MPTP-treated mice	1. Reduce oxidative stress in animal models of PD 2. Release in active form for greater than 24 h	[124]
	AD	Mouse peritoneal macrophages	Solid lipid	Genistein	In vitro: HT22 In vivo: mouse model of AD	1. Delivery functional antioxidant to neuronal mitochondria 2. Cross the BBB and selective target to neurons	[127]
	Encephalitis	monocyte-macrophages	Magnetic nanoparticles	Catalase	In vitro: - In vivo: LPS-induced brain inflammation	1. Target drug to the inflamed brain 2. Deactivate free radicals released by activating microglia	[167]
	Glioma	RAW264.7	DSPE-PEG	IR-792	In vitro: U87L In vivo: orthotopic glioblastoma model	1. Cross BBB and target to tumor site 2. Targeted tumor imaging 3. Combine with PTT and chemotherapy	[168]
	Glioma	RAW264.7	MnO_2_	Cisplatin	In vitro: C6 cells In vivo: orthotopic glioblastoma model	1. Magnetic resonance imaging-guided chemotherapy/chemo-dynamic therapy 2. Good colloidal stability 3. Prolong blood circulation time	[169]
	Glioma	Alveolar macrophages	Gold-silica	-	In vitro: C6 cells In vivo: rat mouse model of C6 cells	1. PTT 2. Effective in vivo in preventing or delaying tumor development	[170]
	Glioma	RAW264.7	Silica	DOX	In vitro: U87MG cells In vivo: U87MG xenograft model	1. Minimally release drug molecules in the early hours of cell entry 2. High tumor accumulation 3. Efficient tumor growth suppression	[171]
	Spinal cord injury	RAW264.7	Liposome	Minocycline	In vitro: inflamed HUVECs In vivo: Spinal cord injury model	1. Actively targeted delivery 2. Decrease cellular uptake in RAW264.7 immune cells 3. Strengthen binding to damaged endothelial cells 4. A comprehensive therapeutic effect	[132]
		RAW264.7	-	Nerve growth factor	In vitro: PC12 In vivo: oxidative stress model of PC12 cells	1. Effectively cellular uptake by PC12 cells and suppression of neuronal apoptosis 2. Good targeting capacity 3. Good behavioral and histological recovery effects	[131]
	Acute ischemic stroke	Primary macrophage	MnO_2_	Fingolimod	In vitro: SH-SY5Y cells In vivo: rat model of transient middle cerebral artery occlusion/reperfusion	1. Actively accumulation in the damaged brain 2. Promote the transition of M1 microglia to M2 microglia 3. Reverse the proinflammatory microenvironment and reinforce the survival of damaged neuron 4. Imaging	[133]
Immune disease	Rheumatoid arthritis	RAW264.7 derived exosomes	-	miRNA	In vitro: HEK-293 T cells and RAW 264.7 In vivo: collagen-induced arthritis model	1. Attenuate inflammation and angiogenesis 2. Inhibit the expression of HNF4A to activate the JAK/STAT3 signaling pathway	[136]
		RAW264.7 derived exosomes	PLGA	-	In vitro: HUVECs In vivo: collagen-induced arthritis model	1. Enhanced targeting effect in vivo in collagen-induced arthritis 2. Bind some RA-promoting cytokines 3. Good biocompatibility	[139]
		KG-1 macrophages	Silicon	-	In vitro: EA.hy 926, HEK-293 and hEpG2 In vivo: -	1. Prolong circulation time 2. No activating of immune system 3. Attenuate the immune-stimulative potential of particles	[138]
		RAW264.7 derived M2 exosomes	-	Plasmid DNA; betamethasone sodium phosphate	In vitro: RAW264.7 In vivo: collagen-induced arthritis model	1. Promote macrophage polarization 2. Good accumulation at inflamed joint sites 3. High anti-inflammatory activity 4. Non-toxic both in vitro and in vivo	[137]
		RAW264.7	ZIF-8	Dexamethasone	In vitro: RAW264.7 In vivo: Collagen-induced arthritis model	1. High drug loading and encapsulation efficiency 2. High stability 3. Long circulation time 4. Sustained drug release in inflamed joint tissues	[65]
		Peritoneal macrophages	PLGA	Dendrobium polysaccharides	In vitro: RAW264.7 In vivo: vaccinated mice and restimulated with ovalbumin	1. Promote antigen uptake by macrophage and lymphocyte proliferation 2. Increase the expression of MHC II, CD80 and CD86 3. Upregulate the ratio of CD4^+^ to CD8^+^ T cells in immunized mice	[172]
Others	Osteoarthritis	Alveolar macrophage cell	Gold	-	In vitro: cartilage explants In vivo: -	1. Superior efficacy in sponging the pro-inflammatory cytokines 2. Alleviating OA inflammation and matrix degradation 3. Enhanced therapeutic efficiency	[144]
	Allergic asthma	BMMs	PLGA	Dnmt3aos	In vitro: M2 macrophage In vivo: allergic asthma mice	1. Ameliorate allergic asthma with a marked reduction of lung inflammation 2. Retain over 48 h and target m2 macrophages 3. No obvious immune function suppression of host	[143]
	Ulcerative colitis	RAW264.7	βCyclodextrin	Rosiglitazone	In vitro: BMDM and Caco-2 cells In vivo: dextran sulfate sodium salt -induced colitis mice model	1. Effectively polarized macrophage to M2 2. Protect epithelial cells from oxidative stress 3. High targeting efficiency 4. Significant therapeutic effects in vivo	[39]
		RAW264.7	MOFs	Carbon nanodots and plasmid	In vitro: colon-26 cells and RAW 264.7 In vivo: dextran sulfate sodium salt-induced UC model	1. Scavenge ROS effectively 2. pH-responsive, immune escape, and inflammation targeting 3. Reduced the expression of proinflammatory cytokines	[141]
	Kidney	RAW264.7 derived exosomes	-	Dexamethasone	In vitro: - In vivo: LPS- or ADR-induced renal inflammation and fibrosis	1. Effectively delivered into inflamed kidney 2. Significant anti-inflammatory efficacy 3. Significantly attenuated renal injury	[140]
	Acute pancreatitis	J774A.1	PLGA	-	In vitro: J774A.1 macrophages In vivo: acute pancreatitis mouse model	1. High biocompatibility 2. Effective protect against disease-associated inflammation, tissue damage and lethality	[142]

### Target delivery in cancer therapy

Considering the role of macrophages in the tumor microenvironment, macrophages can target tumors and phagocytose cancer cells. Macrophage membranes bioinspired nanocarriers have exhibited many advantages such as prolonged circulation and specific target tumor tissue. Currently, macrophage membrane-coated nanoparticles have shown unique essential therapeutic effects in cancers, including breast, skin, lung, colon and others.

#### Breast cancer

Breast cancer has already become a worldwide public health problem for women. Macrophages are the main cellular components of the breast tumor microenvironment and play an important role in the development and metastasis of tumors. Zhang et al*.* have developed natural macrophage membrane-coated paclitaxel-loaded nanoparticles (cskc-PPiP/PTX@Ma) to exhibit an enhanced therapeutic effect for the treatment of primary breast cancer [67]. Cskc-PPiP/PTX@Ma showed higher accumulation at the tumor site because the macrophage membrane with its associated membrane protein served as a concealing cloak against RES clearance and a tumor-homing navigator. For the treatment of breast cancer metastasis, Sun et al*.* created a polymer of PLGA that was used to encapsulate saikosaponin D to formulate biomimetic polymer nanoparticles coated by macrophage membranes hybridized with T7 peptide (SCMNPs) [68]. The T7 peptide is a targeting ligand for transferrin receptors that are overexpressed in tumor cells. The in vitro cellular uptake and in vivo biodistribution studies showed that SCMNPs could specifically target tumor cells and exhibited an immune escape effect on RES. In vivo antitumor performance demonstrated that SCMNPs significantly inhibited tumor growth compared with the control groups. Pretreated macrophage membranes are also used for the encapsulation strategy. For example, Cao’s group synthesized macrophage membrane-coated emtansine liposomes (MEL) to target metastatic foci in the lung [35]. The macrophage membranes were isolated from RAW264.7 cells with high expression of α4 and β1 integrins, which can specifically bind to VCAM-1 on cancer cells. An in vitro cell uptake study showed a 2.0-fold higher internalization into 4T1 cells by FACS than into emtansine liposomes but a lower internalization into RAW264.7 cells. The in vivo pharmacokinetic study displayed the intensity of fluorescence signals of MEL was much higher than emtansine liposome at 1.0 and 4.0 h after administration. Meanwhile, the in vivo distribution of MEL showed a higher fluorescence signal in the lung than in other organs owing to the high expression of α4 and β1 integrins on the macrophage membranes.

A previous study confirmed that M1 macrophages had the natural ability to migrate into tumor tissues and secrete proinflammatory factors [69]. Furthermore, Zheng et al*.* found that exosomes secreted from the macrophage RAW264.7 by LPS can induce neuroprotective function after ischemic stroke by enhancing the anti-inflammatory M2 phenotype polarization of microglia [70]. Wang et al*.* used M1 exosomes loaded with paclitaxel (PTX-M1-Exos) to enhance the antitumor activity of breast cancer [71]. The results show that M1 exosomes produced proinflammatory cytokines and enhanced the antitumor efficiency. After loading the chemotherapeutic agent paclitaxel, PTX-M1-Exos exhibited a higher antitumor effect than M1-Exos alone.

Currently, some investigators transfer their attention to macrophage hybrid membrane biomimetic nanoparticles. For example, Gong et al*.* developed macrophage-4T1 hybrid membrane-camouflaged PLGA loaded with the FGL1/LAG3 blockade molecule siFGL1 and the immune-metabolic adjuvant metformin [72]. The hybrid membrane made use of the homologous targeting capacity of cancer cells and the ability of immune escape of macrophages to realize precise targeting. All studies showed that macrophage membranes and macrophage-derived membrane-coated nanoparticles have great potential in the treatment of primary and metastatic tumors.

Phototherapy is emerging as a new therapeutic strategy for the treatment of cancer because of its noninvasiveness, high selectivity and low systemic toxicity, such as the well-known photothermal therapy (PTT) and photodynamic therapy (PDT) [73]. For PTT, Xuan et al*.* designed macrophage cell membrane-coated gold nanoshells (MPCM-AuNSs) that exhibited good colloidal stability and maintained the original NIR adsorption of AuNSs [74]. The formulation showed higher accumulation at the tumor site. By incubating FITC-dextran-loaded bare AuNSs and MPCM-AuNSs with 4T1 mouse breast cancer cells, 83.18% of the MPCM-AuNSs were internalized into 4T1 cells, whereas only 42.15% of the AuNSs were internalized. The pharmacokinetic studies showed that MPCM-AuNSs had significantly enhanced blood retention time compared to the bare AuNSs. Upon NIR laser irradiation, local heat generated by the MPCM-AuNSs achieves high efficiency in suppressing tumor growth and selectively ablating cancerous cells within the illuminated zone. For PDT, Chen et al*.* reported tumor-associated macrophage membranes (TAMMs) coated with UCNPs (NPR@TAMM) for the treatment of cancer and improving immunotherapy [75]. TAMMs were derived from purified primary TAMs, which highly expressed specific protein markers, including CSF1R, CD206, F4/80, and CD11b. The membranes had the ability to deplete the CSF1 secreted by the tumor cells in the TME, resulting in blockade of the interaction between TAMs and cancer cells. After irradiation with a 980 nm laser for 5 min, NPR@TAMMs inhibited cell proliferation in 4T1 cells with an IC50 value of 59.7 μg/mL, which was significantly lower than that for NPR (301.5 μg/mL) or NPR@MMs (129.2 μg/mL). The TAMM coating strategy allowed the core nanoparticle to be a passive and active target to the tumor while avoiding oxidation and invasion by the immune system. Based on the great effects of chemotherapy, PTT and PDT, researchers have combined all three. Poudel et al*.* established a nanoplatform (PTX@CuS@MMNPs) containing three therapies to kill tumor cells [33]. CuS was used as a nanocarrier for chemotherapeutic drugs, PTT and PDT. Cellular internalization was improved by membrane encapsulation. In vivo tumor accumulation, tumor inhibition rate, and apoptotic marker expression were significantly improved.

The above examples demonstrate that macrophage membrane macrophages and macrophage-derived membrane-coated phototherapeutic agents have the ability to target tumors, escape immune responses and treat breast cancer. Combined with phototherapy, these synergistic systems achieve a high-precision treatment of tumors and an excellent treatment efficiency.

#### Skin cancer

**Fig. 4:**
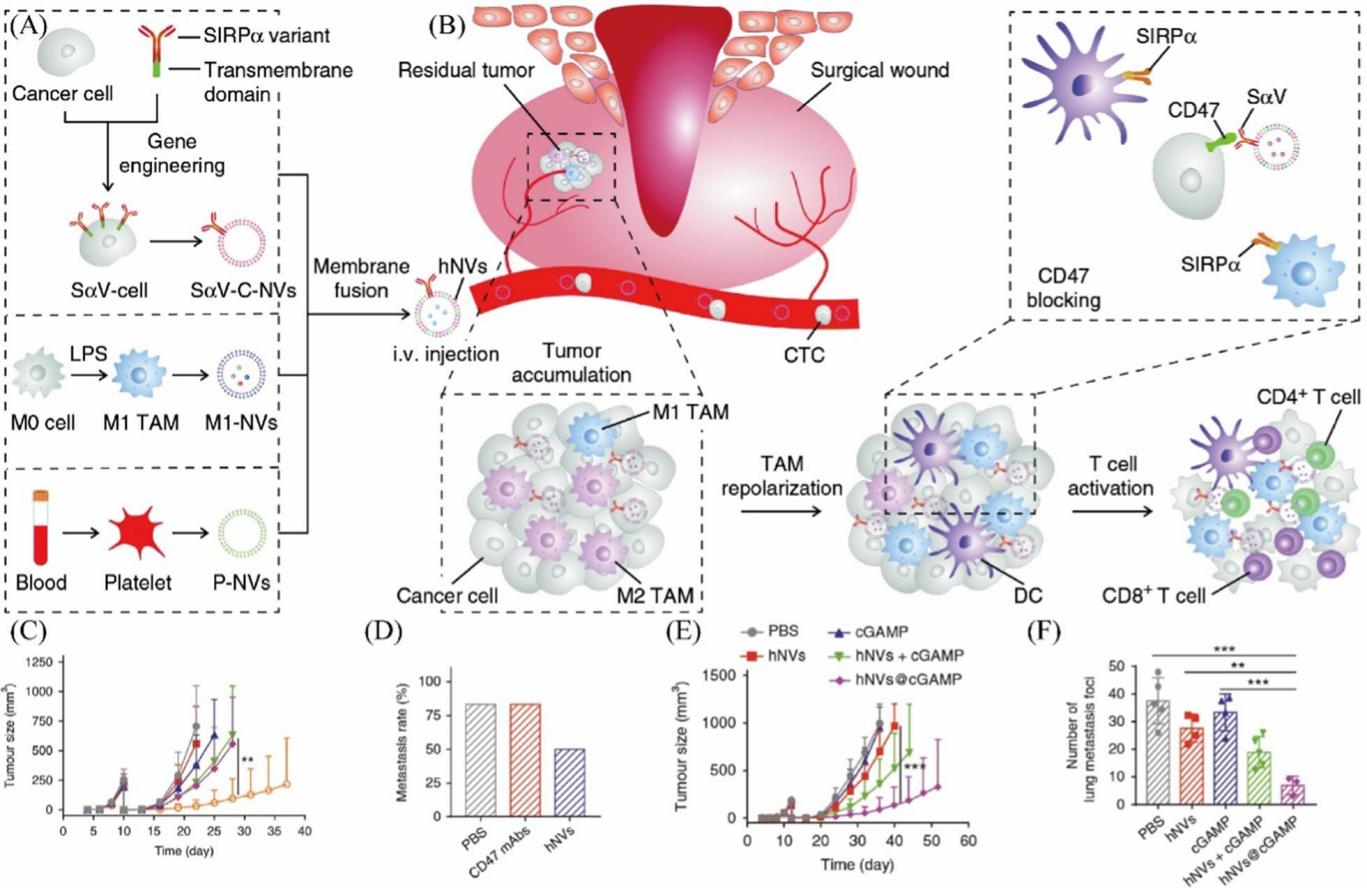
**A** Schematic showing the hNVs consist of engineered SαV-C-NVs, M1-NVs, and P-NVs. **B** Schematic showing the hNVs efficiently interact with CTCs in the blood, accumulate in the post-surgical tumor bed, repolarize TAMs towards M1 phenotype, and block the CD47-SIRPα ‘don’t eat me’ pathway, thus promoting macrophage phagocytosis of cancer cells, as well as boosting antitumor T cell immunity. Average **C** tumor growth kinetics in 4T1 tumor groups. **D** Metastasis rates after indicated treatments. Average **E** tumor growth kinetics in B16F10 tumor groups. **F** Numbers of lung metastatic foci after different treatments. Adapted with permission from [81], copyright ^©^ 2022 Springer Nature Limited

Skin cancer is a malignant tumor due to subcutaneous tissue lesions. Macrophages play a crucial role in the development and migration of skin cancer. Studies have shown that macrophages in squamous cell carcinoma (SCC) can indirectly inhibit T-cell activity by expressing arginase-1, which breaks down L-arginine [76, 77]. Chen et al*.* reported macrophage membrane-coated nanoparticles to increase the circulation time and targeting ability in a model of SSC-7 tumor-bearing mice [54]. Additionally, macrophages can secrete periostin and thus promote the metastasis of melanoma [78]. Melanoma is a kind of aggressive skin cancer that originates from melanocytes. The current limited available treatment options for melanoma are due to its high metastasis rate. Parodi et al*.* have shown that leukocyte membrane biomimetic nanoparticles enhance circulation time and tumoritropic accumulation in mice with murine B16 melanoma [79]. Furthermore, Cao et al. developed albumin nanoparticles coated with macrophage membranes loaded with paclitaxel to treat melanoma [80]. Albumin nanoparticles can accumulate in the tumor site, and macrophage membranes can reduce the clearance of albumin by RES and enhance the target capacity to tumor. Melanoma is an aggressive tumor. Even though it works well on primary tumors, there is no better treatment for cancer cells in the circulation. Cancer cells form circulating tumor cells (CTCs) by binding to immune cells, including macrophages and platelets, thus avoiding the clearance of the immune system and low efficiency of metastasis. Rao et al*.* developed hybrid nanovesicles (hNVs), including M1 macrophage membranes, platelet membranes and cancer cell membranes, that overexpressed high-affinity SIRPα variants by loading a stimulator of interferon genes (STING) agonist for the treatment of B16F10 tumors and 4T1 tumors (Fig. 4A, B) [81]. The hybrid nanovesicles increased the affinity for CD47 because they overexpressed SIRPα and promoted the M2-to-M1 repolarization of macrophages. Notably, researchers have used six nanovesicles, including liposomes, red blood cells, platelets, M1 macrophages, engineered cancer cells and hNVs, for comparison. The in vivo pharmacokinetic study and fluorescence imaging showed that M1-NVs exhibited a longer blood retention time and more significant accumulation at the tumor site in the B16F10 mouse melanoma model. The mRNA levels of M2 macrophages after treatment with M1-NVs demonstrated a lower level of M2 markers and a higher level of M1 markers. As a result, M1-NV treatment induced a significant increase in tumor-infiltrating T cells, especially CD8^+^ T cells, effectively improving the antitumor effects. According to B16F10 mouse melanoma models, hNVs significantly suppressed local recurrence and distant metastasis of tumors (Fig. 4C, D). Due to the suppression of tumor recurrence, the survival rate of the mouse groups treated with hNVs increased to 66% in 60 days. Additionally, hNVs demonstrated a higher inhibition of 4T1 tumors in a poorly immunogenic triple-negative breast cancer model (Fig. 4E, F). Remarkably, the platelet-derived NVs suggested the damaged tissue targeting capability and effective binding of pNVs with CTCs.

The most critical aspect of the capture for CTCs is that the researchers have combined the advantages of the three cells. Platelet and macrophage membranes evade recognition by the immune system, thus allowing high-affinity cancer cell membrane variants to target tumors more precisely.

#### Lung cancer

Lung cancer is characterized by the uncontrolled proliferation of cancer cells in the lung. Meanwhile, the lung is the most common site of metastasis and tumor recurrence, which is the leading cause of cancer-related death. Non-small cell lung cancer accounts for > 80% of lung cancer cases and currently has an overall 5 year survival rate of only 15%. Evangelopoulos et al*.* have made use of electroporation to load doxorubicin into J774.1 macrophages; thereby, the system can deliver the drug into the pulmonary environment or lungs by its tropism for neoplastic and release drug through efflux bump [82]. In the LL/2 mouse model, this system has demonstrated favorable suppression of tumor growth in the lungs. Notably, the cell viability of macrophages is inactive, and previous work showed that electroporated cells maintain the expression of adhesion-based transmembrane proteins. With the application of electroporation, they have developed a low-cost and high-efficiency solution to generate effective pulmonary drug delivery vehicles. The aforementioned macrophage membranes are not focused on the phenotype of macrophages. According to a report, non-small cell lung cancer overexpresses the sigma receptor, which is a membrane-bound protein [83]. Based on this finding, Kim et al*.* prepared PTX-loaded exosomes modified with aminoethylanisamide-polyethylene glycol (AA-PEG) to target pulmonary metastases [84]. Exosomes are harvested from the supernatants of RAW264.7 cells and primary bone marrow-derived macrophages to enhance the blood circulation time and tumor accumulation rate. In vivo therapeutic efficacy was greater than that of nonvectorized exosome-PTX and Taxol. Choi et al*.* used mouse peritoneal macrophages induced by 3% Brewer thioglycollate medium as a biocarrier [85]. They provided liposome-loaded doxorubicin and the imaging agent iron oxide, which can reach the tumor site in vivo, maintain continuous and durable action in tumor death and exhibit imaging capacity.

#### Others

Apart from the above types of cancer, macrophage membrane-mimetic nanoparticles are also being applied to other cancers. For example, Fang et al*.* prepared artificially assembled macrophages with drug-loaded liposomes for colorectal cancer [86]. Integrin α4, integrin β1 and C-C chemokine receptor 2 (CCR2) help them target tumors and evade the mononuclear phagocyte system. He et al*.* designed polyelectrolyte multilayer (PEM) capsules coated with gold nanoparticles and THP-1-cell membranes on the two sides to be used as photoactive cancer cell detectors to kill HeLa cells by PTT [62]. Upon illumination with NIR light, the gold shell could be heated to evaporate water violently and melt the capsule and cellular walls. In vitro experiments of biofunctionalized Janus capsules indicated that they can successfully kill cancer cells with a laser power density of 23.6 mW/μm^2^. Qiang et al*.* designed reduced graphene oxide-loaded doxorubicin, followed by internalization of macrophages to work on RM-1 mouse prostate cancer [87]. Ji et al*.* created hybrid membranes, including H22 cells, HepG2 cells and RAW264.7 cells, to target hepatocellular carcinoma [88].

Overall, the aforementioned studies indicate the potency of macrophage membrane-cloaking nanoparticles in the treatment of cancers. Macrophage membrane coating does not influence the function of the core but endows the core with targeting and immune evasion. Additionally, macrophage membrane camouflage nanoparticles can steadily improve the solubility and target capability of drugs. This strategy can also utilize the membrane protein to overcome the challenge faced by nanoparticles during systemic circulation. Macrophage membrane- or membrane-derived exosomes can be edited and modified. Notably, macrophages play important roles in tumor metastasis. A previous study showed that tumor macrophages promote epithelial-mesenchymal plasticity to fit the shear stress in blood vessels [89]. Based on this mechanism, macrophages have a potent ability to kill CTCs, such as leukemia, multiple myeloma and malignant lymphoma. Taking into account the role of macrophages, macrophages have natural superiority in cancer treatment.

### Anti-microbe

Pathogenic microorganisms refer to microorganisms, or pathogens, that can invade the human body and cause infections or even infectious diseases. Among the pathogens, bacteria, viruses and parasites are harmful. The membranes from immune cells, including macrophages, have a mass of bacterial-specific cellular receptors that allow the nanoparticles to attract and neutralize the membrane active toxins. This phenomenon is called the ‘nanosponge effect’ due to its high affinity for toxins and cytokines [90].

#### Antibacterial

With the discovery of penicillin, antibacterial agents have been emerging as a new therapeutic strategy. However, the emergence of bacterial resistance to antibacterial agents has increased the use of antibiotics in clinical treatments for infection, mainly because of three biological properties: cell envelope blockages, biofilm protection, and macrophage shelter [91, 92]. Indeed, macrophages can specifically recognize and neutralize bacteria through the connection between PRRs on the macrophage membrane and PAMPs of bacteria. For example, in sepsis, endotoxin, referred to as lipopolysaccharide (LPS), is released from the bacteria and is recognized as a PAMP by macrophages [93]. Subsequently, macrophages can bind and neutralize endotoxins and cytokines. Soracha et al*.* prepared a PLGA core coated with a macrophage membrane (MΦ-NPs) for the treatment of sepsis [94]. MΦ-NPs could bind to endotoxins and cytokines, inhibiting their ability to enhance downstream inflammatory cascades. In a mouse *Escherichia coli* bacteremia model, treatment with MΦ-NPs significantly reduced proinflammatory cytokine levels and inhibited bacterial dissemination. In another study, scholars found that macrophage-derived biomimetic nanoparticles (leukosomes) could be used for the treatment of an LPS-induced murine model of sepsis [95]. In vitro studies elucidated that leukosomes induce an anti-inflammatory response in endothelial cells through the interaction of leukosomes with macrophages. The interaction could trigger a decrease in proinflammatory genes (IL-6, IL-1b, TNF-α) and an increase in anti-inflammatory genes (IL-10, TGF-β).

Interestingly, when macrophages were cultured with specific bacteria, the expression of recognition receptors, such as TLR2, TLR4 and TLR6, increased. Toll-like receptors function as PRRs to produce an immune response. Wei et al*.* reported a nanotoxoid formulation for use as a vaccine against gram-negative bacterial infections by a multiantigenic formulation [96]. This formulation was first performed on PLGA core-coated J774 macrophage cell membranes with a weight ratio of 1:1, followed by sonication. Then, they collected P. aeruginosa secretions (PaS) from the bacterial culture supernatant and incubated them with macrophage membrane-coated nanoparticles for 15 min to obtain the final macrophage nanotoxoids (MΦ-toxoids). MΦ-toxoids endowed the nanoparticles with multiantigenic characteristics to clear bacteria. Likewise, on account of this phenomenon, this group used a pretreated macrophage membrane to coat a gold-silver nanocage (Sa-M-GSNCs) to more precisely target bacterial cell surfaces [97]. GSNC has been approved as an effective antibacterial agent because of its photothermal effect. Both in vitro and in vivo live bacterial infection of cells and mice showed that Sa-M-GCNCs had a better therapeutic effect. Li et al*.* regarded the use of bacterially pretreated macrophage membranes as highly impractical for intracellular bacterial infections [98]. They designed a self-assembled micelle composed of antimicrobial triclosan and ciprofloxacin coated with macrophage membranes (Me-ANPs) to target infected macrophage cells where bacteria hide. Both in vitro and in vivo showed efficiency in killing *S. aureus*. Me-ANPs eradicated the infection in a mouse peritoneal infection model and mouse organ infection model.

#### Antiviral

A virus is a much smaller microbe than fungi and bacteria that relies on the metabolic system of host cells for reproduction. Infected cells often die because the virus blocks their normal physiological functions, subsequently resulting in the release of new viruses to infect other cells. The mechanism of action of antiviral infection drugs is mostly to interfere with the replication of the virus [99]. However, when antiviral drugs enter the body, in addition to being able to attack viruses and infected cells, they can also produce certain toxicity to normal cells in the human body [100]. The antiretroviral delivery system is an effective measure among antiviral drugs currently on the market. However, there are still three problems: weak antiviral effects, serious side effects, and rapid drug resistance.

**Fig. 5:**
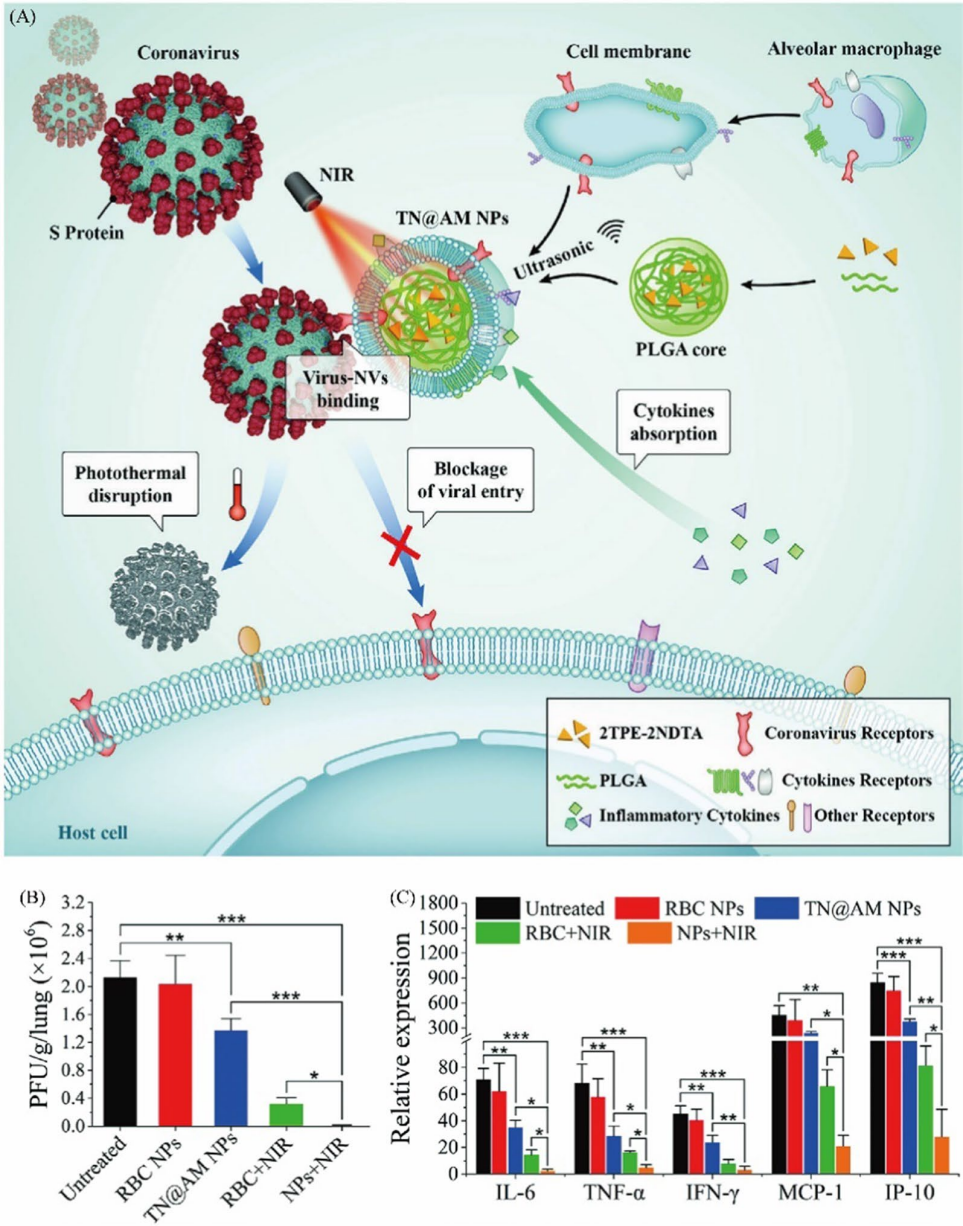
**A** Schematic illustration of multifunctional alveolar macrophage-like nanoparticles for coronavirus cellular entry blockage, virus photothermal disruption, and inflammatory cytokines absorption. Physicochemical and membrane protein characterizations of TN@AM NPs **B** Detection of MHV-A59 burden in the lung tissues through a standard plaque assay at 5 days post different treatments. **C** Detection of the mRNA expression of various proinflammatory cytokines in the lung tissues through a standard RT-qPCR assay at 5 days post different treatments. Adapted with permission from [36], copyright ^©^ 1999–2022 John Wiley & Sons, Inc.

Dou et al. developed a nanoparticle-loaded indinavir (NP-IDV) formulation packaged into carrier bone marrow-derived macrophages (BMDMs) for human immunodeficiency virus type 1 (HIV-1) [56]. Macrophage membrane protein endowed NP-IDV with the ability to target sites, promoting sustained “local” drug release for periods of approximately 2 weeks, avoiding the destruction of the immune system in the HIV-1 mouse model and increasing the therapeutic effects. In addition, they used IDV-NP-BMM to target HIV-1 encephalitis (HIVE) rodent model [101]. The results showed continuous IDV release for 14 days and a reduction in HIV-1 replication in HIVE brain regions. Similarly, severe COVID-19 was highly associated with cytokine storm syndrome (CSS). Tan et al*.* designed macrophage membrane-coated PLGA NPs loaded with lopinavir (PLGA-LPV@M) [102]. The receptors of IL-1β and IL-6 on the macrophage membrane neutralize proinflammatory cytokines to suppress macrophages and neutrophils. Moreover, macrophages expressed angiotensin-converting enzyme 2 (ACE II), which could help PLGA-LPV@M target SARS-CoV-2 by the affinity between ACE II and the spike protein on SARS-CoV-2. Owing to the synergistic effects, PLGA-LPV@M exhibited a significant effect in the mouse model of coronavirus infection. Li’s group reported that alveolar macrophages (AMs) were the first line of defense for the host immune system against SARS-CoV-2 infection [36]. Therefore, an AM membrane was used to coat PLGA nanoparticles embedding photothermal material (TN@AM NPs) to enhance antiviral efficiency (Fig. 5A). In a surrogate mouse model of COVID-19, TN@AM NP treatment decreased the lung virus burden compared to the untreated group (Fig. 5B). Anti-cytokine ability analysis demonstrated that the TN@AM NP group significantly decreased the expression of various proinflammatory cytokines (Fig. 5C). Furthermore, lung histopathological alteration analysis showed that TN@AM NPs significantly reduced lung damage compared to the other NPs.

#### Anti-parasite

Macrophages also play an important role in parasite infection, participating in parasite-host immune interactions [103]. A study confirmed that parasites can induce M1-type polarization of macrophages. M1 macrophages clear parasites by producing TNF-α, IL-12 and iNOS. M2 macrophages can metabolize L-arginine into proline and polyamine to promote parasite survival through Arg1, which is related to the inflammatory process and regression of tissue repair [104]. *Leishmania donovani*, an intracellular parasite, adheres to macrophages and enters into macrophage cells along with phagocytosis activity to induce transformation to the M2. Amphotericin B has been encapsulated into macrophage nanogghosts to *Leishmania*-infected macrophages [105]. It has been shown that an increased concentration at the infected site and the production of protective cytokines and ROS-RNS with lower collateral toxicity to healthy cells compared to the free amphotericin B group.

Taken together, these results showed the ability of macrophage membrane-coated nanoparticles to regulate the inflammatory response in target cells, acting as a bioactive nanotherapeutic.

### Cardiovascular diseases

Atherosclerosis (AS) is a chronic inflammatory vascular disease caused by the accumulation of many lipids around the arterial wall. As an oxidized form of low-density lipoprotein (Ox-LDL) in the intimal layer, the local endothelial wall produces excessive ROS and subsequently secretes a series of adhesion molecules to recruit immune cells [106]. Subsequently, local inflammation leads to fatal cardiovascular events and stroke by stenosis of the arterial lumen and rupture of unstable plaques [107]. Integrin α4 and β1 are major leukocyte receptors on the macrophage membrane that recruit macrophages to atherosclerotic plaques by recognizing VCAM-1 on the surface of vascular endothelial cells [108, 109].

Fe_3_O_4_ has been used as a typical magnetic resonance imaging (MRI) agent. Huang et al*.* confirmed that Fe_3_O_4_ nanoparticles coated with a RAW264.7 membrane (Fe_3_O_4_@M) could effectively target the early process of AS [110]. Compared with the conventional MR agent, Fe_3_O_4_@Ms had more safety for research objects in vivo or in vitro and were more effective in imaging than Fe_3_O_4_@PEG. The molecular mechanism study showed that integrin α4 and β1 overexpression on macrophages specifically recognized VCAM-1 on endothelial cells. Wang et al*.* fabricated a macrophage membrane coating on the surface of rapamycin-loaded PLGA (MM/RAPNPs) to target atherosclerotic lesions [111]. The release kinetics showed that 38.51% and 35.62% of rapamycin were released from RAPNPs and MM/RAPNPs after 72 h of incubation, respectively. MM/RAPNPs approved sustained drug release. Cell phagocytosis assays were performed to evaluate immune evasion. Both CLSM images and FACS analysis showed that macrophage membrane-coated DID-labeled NPs (MM/DIDNPs) could significantly inhibit internalization by macrophages. Similarly, an overexpression VCAM-1 model of HUVECs activated by TNF-α demonstrated a higher internalization compared with DIDNPs. Rapamycin is practically insoluble in water, resulting in low bioavailability. The in vivo therapeutic efficacy of MM/RAPNPs showed the lowest atherosclerotic lesions in 6.59% compared with 18.3% of free RAP and 14.43% of RAPNPs. ORO and toluidine blue further verified the therapeutic efficacy of MM/RAPNPs. Overall, MM/RAPNPs can effectively attenuate the progression of AS.

Based on the excessive ROS in the local inflammatory site, researchers have developed a delivery system including ROS-responsive NPs coated with macrophage membranes for AS [112]. For example, amphiphilic oxidation-sensitive chitosan oligosaccharide (Oxi-COS) was chosen as a ROS-responsive material to load atorvastatin (AT-NPs). AT-NPs possessed a drug encapsulation efficiency of 48.3% and a drug loading content of 5.1%. After coating the macrophage membrane by coextrusion and bath sonication, a spherical core–shell structure was observed. From the TEM image and DLS, a single layer of cell membrane wrapped the Oxi-COSNPs, and the diameter of the nanoparticles increased from ~ 204 to 227 nm. Considering the key role of foam cells in AS, researchers selected LPS-induced inflammation in macrophages and oxLDL-treated macrophages as in vitro models to evaluate the therapeutic efficacy of MM-AT-NPs. The results revealed the ROS responsiveness of AT-NPs and MM-AT-NPs in vitro, as well as their attenuation. After incubation with macrophages, MM-AT-NPs provided a potential targeted capacity to inflammatory macrophage cells and foam cells, except for the remaining macrophage cells. For the in vivo targeting capability of MM-NPs and NPs/Mas to atheromatous plaques, cyanine 7.5 NHS ester was employed to prepare Cy7.5-labeled formulations. The aortic tissues from MM-Cy7.5-NPs had stronger fluorescence due to their inherent immune propensity for inflammation. Next, they examined the therapeutic effect through a model of female ApoE^−/−^ mice. There was an obvious improvement in plaque area with the treatment of MM-AT-NPs in comparison with AT-NPs, which decreased to ~ 8% of the total aorta tissue area. Interestingly, Ahn et al*.* designed a formulation by encapsulating Ce6 within the triple-helix structure of Glu in aqueous solution [113]. Then, macrophages derived from foam cells internalized the Glu/Ce6 nanocomplexes and delivered nanocomplexes to treat atherogenesis. With laser irradiation, the Glu/Ce6 nanocomplexes significantly damaged the foam cell membranes and generated ROS to reduce ICD. In another article, boronic ester-modified dextran was used to load rapamycin (MM/RNPs) [114]. The average hydrodynamic diameter of MM/RNPs was 164.7 nm. The cumulative release of rapamycin with or without H_2_O_2_ was lower than that of RNPs due to the coating membrane. However, MM/RNPs still have ROS responsiveness to H_2_O_2_. An in vitro uptake assay demonstrated that MM/RNPs could effectively escape from macrophages and inflamed endothelial cells. Zebrafish were used as an animal model to confirm the in vivo biocompatibility of MM/PCD NPs. Various concentrations of MM/PCD NPs and time points were applied to embryos from zebrafish, and the live zebrafish suggested good biocompatibility. These results showed a potential candidate for anti-AS applications.

Although the natural macrophage membrane has many specific membrane recognition proteins, researchers can still modify the expression of membrane proteins to suit their own needs for treatment. Li’s team reported a bionic nanoparticle with overexpressed CD47 and integrin α4 and β1 [115]. Firstly, they used endothelin-1 to enhance the expression of integrin α4/β1. Second, the functionalized CD47 plasmid was transferred into macrophages to upregulate CD47. The modified macrophage membrane vesicles were mixed with PLGA NPs loaded with colchicine (MMM/COL NPs), sonicated and extruded. DLS analysis showed that the diameter of MMM/COL NPs increased from ~ 180.76 to 202.02 nm, which is the thickness of the macrophage membrane shell. The fluorescence of macrophage cells treated with MMM/COL NPs was lower than that of cells treated with DID NPs or MM/COL NPs, suggesting that the overexpressed CD47 and integrin α4 and β1 were functional. Additionally, the MMM/COL NP group had significantly enhanced fluorescence of inflamed HUVECs. Furthermore, an in vivo targeting study showed the best accumulation of MMM/Dil NPs in atherosclerotic lesions and the best targetability to atherosclerotic plaques.

**Fig. 6:**
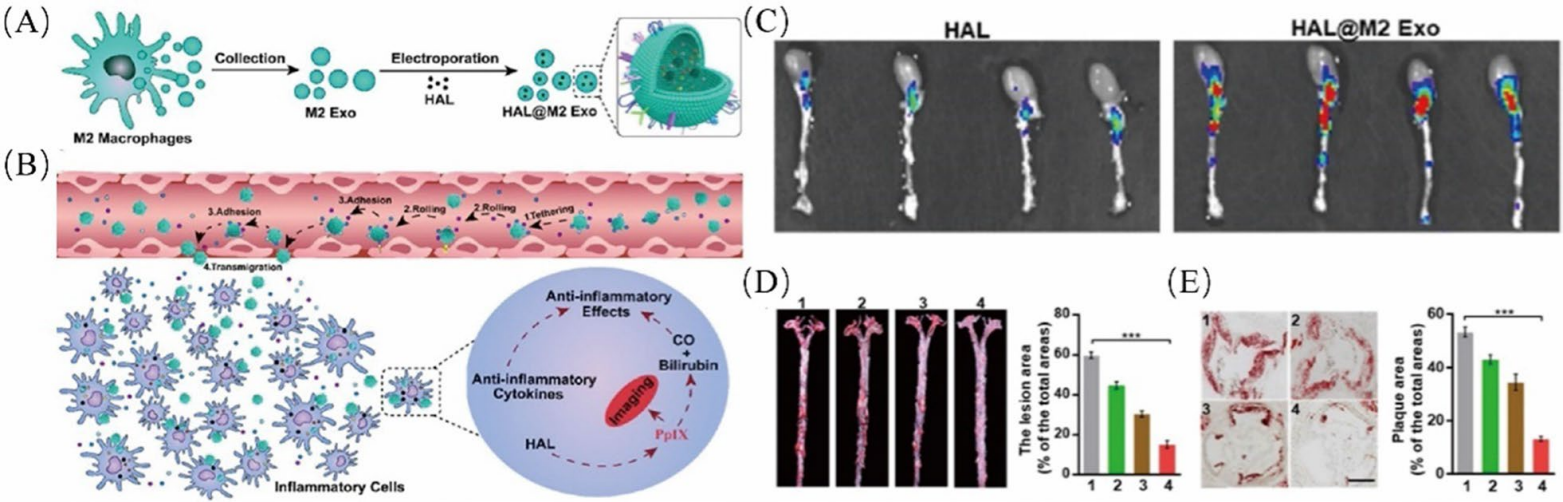
**A** The preparation of HAL@M2 Exo. **B** The inflammation-tropism and anti-inflammation effects of HAL@M2 Exo. Treatment of early atherosclerotic plaques with HAL@M2 Exo. **C** Fluorescence imaging of the aortas excised from mice. **D** Photographs of the excised aortas stained by ORO and the corresponding quantitative analyses of plaque areas. **E** Cryosection photographs of the aortic valves stained by ORO and the corresponding quantitative analyses of plaque areas. Adapted with permission from [116], copyright ^©^ 2020 The Authors. Publishing services by Elsevier B.V

As recently reported, exosomes isolated from macrophages have been applied in AS. Wu et al*.* confirmed and clarified that M2 exosomes had a lower level of proinflammatory factors but anti-inflammatory factors, including IL-10, IL-1Ra, and TGF-β [116]. Hexyl 5-aminolevulinate hydrochloride (HAL) enhanced the anti-inflammatory effect by driving the intrinsic biosynthesis and metabolism of heme, which induced the production of anti-inflammatory carbon monoxide and bilirubin. Next, HAL was taken into M2 Exos via electroporation (HAL@M2 Exos) to treat AS (Fig. 6A, B). DLS analysis revealed HAL@M2 Exos with a diameter of 180 nm and excellent stability after 1 week in PBS. A series of experiments were conducted to determine the mechanism. The expression of adhesion molecules, including E-selectin, VCAM1, ICAM1, CD44, VLA4 and LFA1, on inflammatory endothelial cells was measured. The recognition mechanism led to the excellent transmigration and accumulation of M2 Exos at the inflammation site. The in vivo therapeutic effect of HAL@M2 Exo was evaluated in ApoE^−/−^ mice fed a cholesterol-rich diet. From the fluorescence imaging originating from endogenously biosynthesized protoporphyrin IX (PpIX) in the aortas of mice, the HAL@M2 EXO group exhibited the highest PpIX fluorescence signal (Fig. 6C). Oil Red O (ORO) staining of entire aortas indicated that HAL@M2 Exo observably reduced aortic lesions and aortic valve lesions by 75.2% and 73.9%, respectively, compared with PBS alone (Fig. 6D, E). The H&E staining results showed that the HAL@M2 EXO group had diminished aortic values. Finally, the expression of ABCA-1 and SR-B1 receptors in aortas showed a greatly upregulated tendency, which further confirmed that HAL@M2 EXOs could relieve chronic inflammation-induced AS in vivo.

### Neurological disease

Neuroinflammation is an inflammatory response within the central nervous system and the main hallmark of common neurodegenerative diseases, including Alzheimer’s disease (AD), Parkinson’s disease (PD), and amyotrophic lateral sclerosis (ALS). The main cause of pathogenesis is that hyperactivated microglia release proinflammatory cytokines, which induce neuronal death and accelerate neurodegeneration [117]. However, it is extremely difficult to deliver drugs to microglia. The main obstacle is the blood-brain barrier (BBB), which is difficult for drugs to cross due to its unique and compact physiological structure. It refers to the barrier between blood plasma and brain cells formed by the walls of brain capillaries and glial cells and the barrier between plasma and cerebrospinal fluid formed by the choroid plexus, which can prevent certain substances from the blood from entering the brain tissue [118–120]. Macrophages are naturally capable of crossing the BBB, which can traverse the endothelial wall due to increased margination and extravasation [121].

PD is a neurological disorder. This disease is attributed to the selective loss of neurons in the substantia nigra and is associated with brain inflammation, microglial activation and secretory neurotoxin activities such as ROS [122]. Batrakova et al. have developed a BMM system to deliver catalase to an animal model of PD. Catalase was packaged into a cationic block copolymer, polyethyleneimine-poly(ethylene glycol) (PEI-PEG). It has been shown that the nanoparticle could retain catalytic activity and be released in its active form after 24 h. In vitro experiments have indicated potent antioxidant effects for ROS produced by microglia activated with either N-α-syn or TNF-α. Additionally, an in vivo assay showed that BMM could increase the delivery of labeled enzymes into tissues, including a twofold increase in the number of enzymes in the brains of MPTP-treated mice. Furthermore, this team has explored the pharmacokinetics and biodistribution of BMM-incorporated nanozymes. After coating with a macrophage cell membrane, the BMM-nanozyme processed longer blood circulation than bare nanozyme and more precisely targeted diseased sites in models of PD [123, 124]. A previous study proved that exosomes function as source membranes and cross biological barriers, including the BBB. Haney et al*.* developed an exosome-based delivery system to treat PD by loading catalase [125, 126]. These findings indicated that exosomes loaded with catalase efficiently accumulated in neurons and microglial cells in the brain and produced a potent neuroprotective effect. They internalized nanozymes into BMMs to attract neurons and endothelial cells by endocytosis-independent mechanisms. The results showed that nanozyme trafficking in and between cells could facilitate a neuroprotective response and provide insights.

**Fig. 7:**
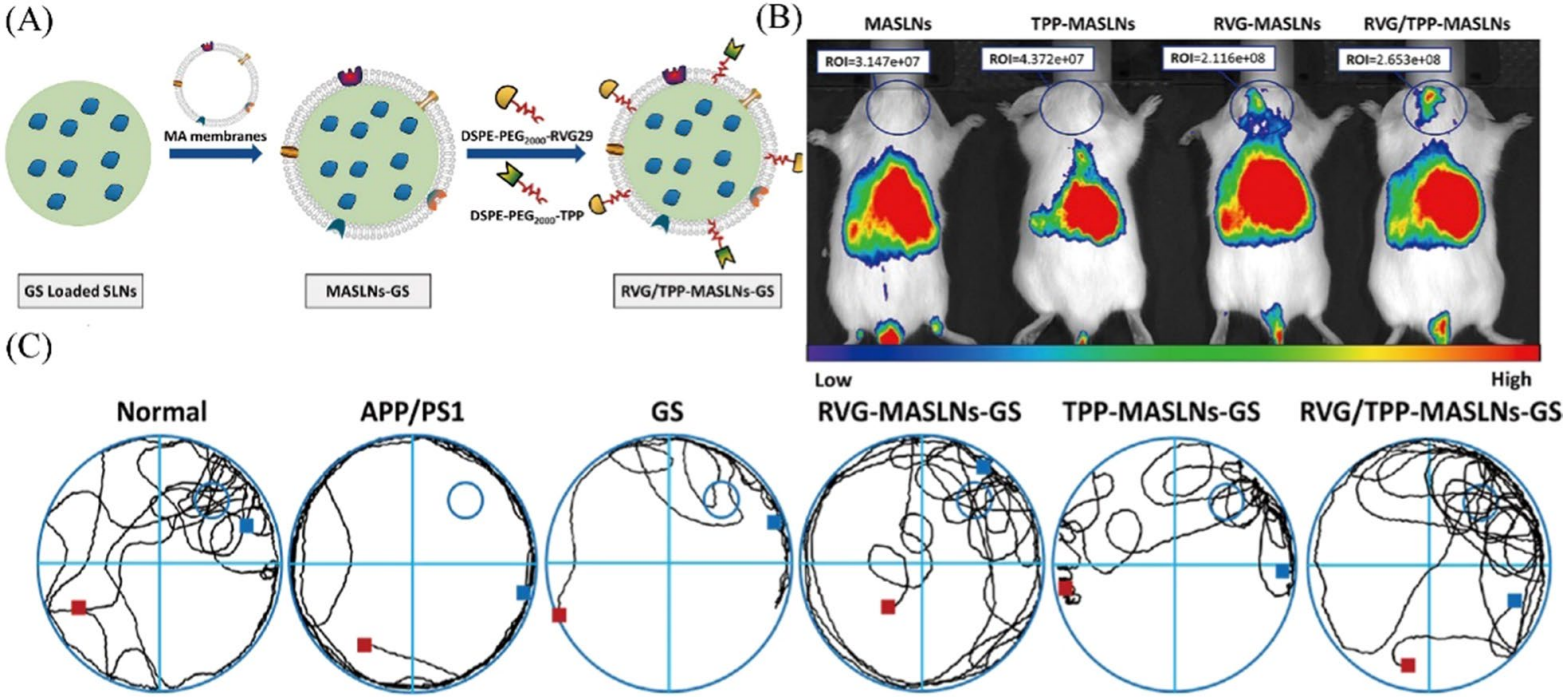
**A** Preparation and characterization of RVG/TPP-MASLNs-GS. **B** In vivo brain-targeting ability. Biodistribution of DIR contained in various formulations determined by IVIS Lumina II. **C** Representative swimming path tracings of different groups. Adapted with permission from [127], copyright ^©^ 1999–2022 John Wiley & Sons, Inc.

New therapeutic targets have been developed for AD, including neuronal mitochondrial dysfunction and aggregation of beta-amyloids. Han et al*.* designed a system in which macrophage membrane-coated solid lipid nanoparticles attached rabies virus glycoprotein (RVG29) and triphenylphosphine cation (TPP) molecules to deliver genistein (GS) (RVG/TPP-MASLNs-GS) to neuronal mitochondria (Fig. 7A) [127]. RVG29 helped the system cross the BBB and target neurons, and TPP further delivered the system to mitochondria via its positive charge. Physical parameters displayed a diameter of 100 nm and a “shell-core” structure. In vivo imaging verified the high brain accumulation of DIR-labeled RVG-MASLNs (Fig. 7B). An in vitro antioxidative stress assay was performed to confirm the protective effect of RVG-MASLN. H&E staining of the hippocampal region of AD mice was further verified. Finally, the swimming path tracing of AD mice showed a more significant learning ability Fig. 7C. In conclusion, RVG-MASLN-GS exhibited excellent effects on relieving AD symptoms in vitro and in vivo.

Spinal cord injury (SCI) is also a common neurological disease, including primary injury and secondary injury [128]. Primary injury refers to injury caused by an external force acting directly or indirectly on the spinal cord. Secondary injury refers to spinal cord edema caused by an external force, hematoma formed by hemorrhage of small blood vessels in the spinal canal, compression fracture, and broken intervertebral disc tissue [129]. Secondary injury is the main obstacle to motor function recovery after spinal cord injury. Macrophages are characterized by chemotaxis to inflammatory sites, penetrating blood vessels, and targeting the damaged central nervous system through the interaction of adhesion factors and integrins [130]. Xia’s group encapsulated nerve growth factor (NGF) into the macrophage membrane (NGF-NVs) to treat SCI [131]. Cell viability assays showed that NGF-NVs were effectively taken up by PC12 cells and inhibited neuronal apoptosis. Furthermore, they found that NGF-NVs sharply increased the survival of neurons by activating the PI3K/AKT signaling pathway and had a good effect on SCI. In another article, researchers developed the use of membranes from different macrophage subtypes to coat liposomes loaded with minocycline [132]. All experiments showed that the formulation decreased cellular uptake by macrophages, prolonged drug circulation time and actively targeted the trauma site of SCI.

Regarding neuronal survival in ischemic stroke, reperfusion injury is still a major obstacle. Compared with glial cells and vascular cells, neurons are the most vulnerable cells because of the rapid loss of normal function under ischemic conditions. Li et al*.* developed a macrophage-disguised honeycomb manganese dioxide (MnO_2_) nanosphere loaded with fingolimod (Ma@(MnO_2_ + FTY) [133]. On the one hand, Ma@(MnO_2_ + FTY) crosses the BBB, and MnO_2_ nanospheres can reduce oxidative stress and promote the transition of M1 microglia to M2 by consuming excess hydrogen peroxide (H_2_O_2_). On the other hand, consuming ROS led to the inhibition of the NF-κB signaling pathway in microglia to mitigate the proinflammatory response. Fingolimod can activate the signal transducer and activator of the transcription-3 (STAT3) pathway. The results indicated that Ma@(MnO_2_ + FTY) successfully protected neurons and provided new possibilities for the treatment of brain disease.

Pang et al*.* used primary M1 macrophages as a carrier combined with PLGA nanoparticles to deliver doxorubicin (DOX@M1-NPs) to glioma tumors [134]. The results of the in vivo endothelial barrier model revealed that DOX@M1-NPs significantly boosted transcytosis across the endothelial barrier and uptake by U87 cells. In vivo imaging experiments verified that M1-NPs exhibited significant superiority in brain U87 tumors. The in vivo antiglioma effect showed that DOX@M1-NPs markedly prolonged mouse survival by 38.5 days.

### Immune diseases

Macrophage membrane-camouflaged nanoparticles are used for the treatment of immune diseases. Rheumatoid arthritis (RA) is an autoimmune disease that leads to joint inflammation, resulting in functional disabilities. The pathology of RA mainly involves the proliferation of synovial lining cells, the infiltration of a large number of inflammatory cells in the interstitium, the neovascularization of microvessels, the formation of pannus, and the destruction of cartilage and bone tissue. Evidence has shown that macrophages adhere to the synovium or the pannus of inflamed vascular tissue via specific ligands. In this case, macrophage membranes or macrophage exosome membranes can take the burden of biomimetic nanoparticles to decrease inflammatory cytokines [65, 135–137].

**Fig. 8:**
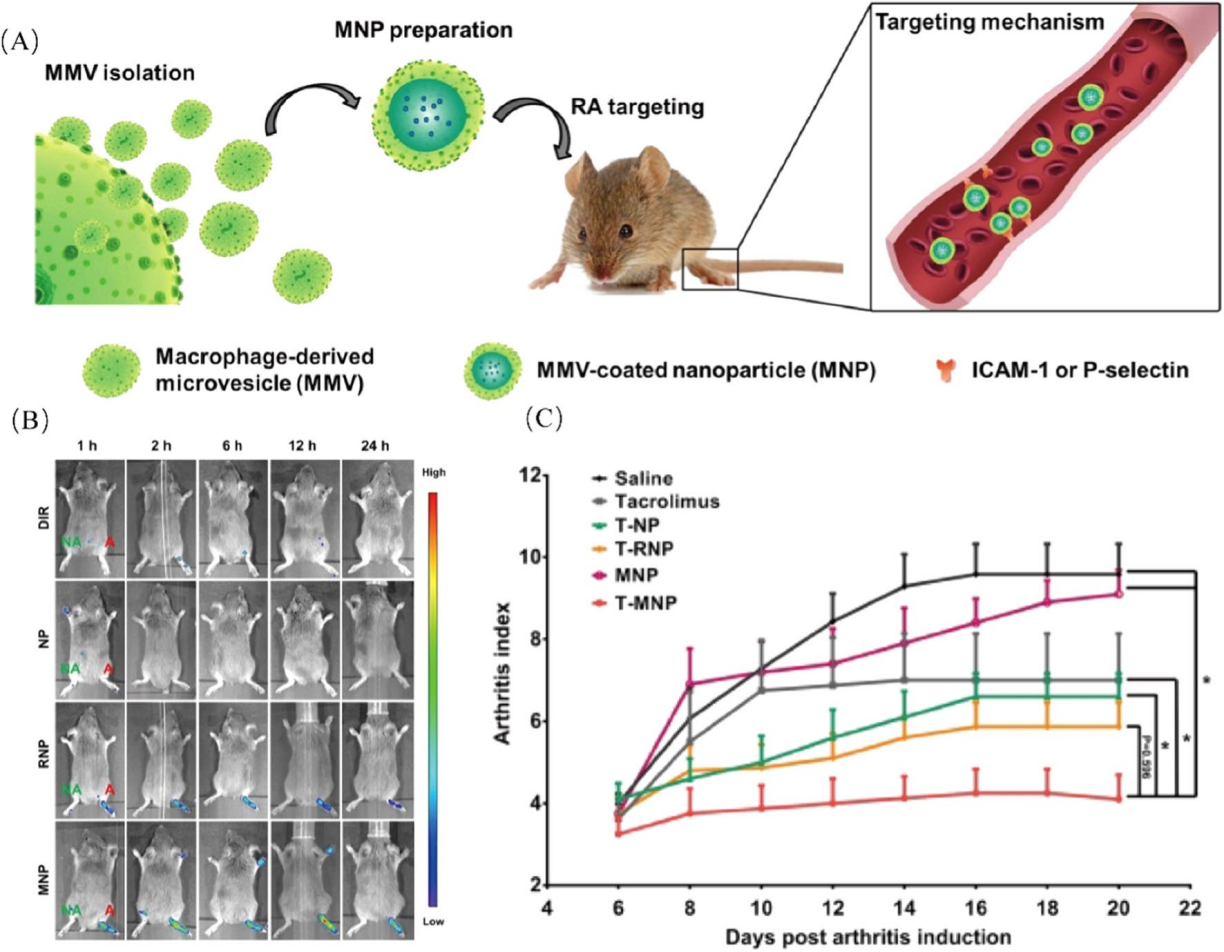
**A** Schematic illustration of MMV-coated nanoparticle (MNP) targeting sites of RA. MNP could target sites of RA through ICAM-1 or P-selectin adhesion. **B** Representative images of different nanoparticles accumulation in arthritic paws or nonarthritic (NA) paws; n = 3. **C** Arthritis index in different groups over 14 days of treatment. Adapted with permission from [139], copyright ^©^ 2022 American Chemical Society

The current treatment of autoimmune disease presents systemic side effects. Flavia et al. developed composite platforms made of porous silicon (Psi) coated with KG-1 macrophage cell membranes (TCPSi@KG-1) for RA [138]. They analyzed the size, surface morphology, and stability in different biological buffers of TCPSi@KG-1, followed by the biological evaluation of cytocompatibility and immunological profile. As a result, TCPSi@KG-1 greatly enhanced the stability of the hydrophobic particles in plasma and simulated synovial fluid. The cytocompatibility of TCPSi@KG-1 in different cell lines, including target organs, blood vessels, kidneys and livers, reached 48 h at concentrations ranging from 0.5 to 50 μg/mL. In addition, the immunological profile investigated in KG-1 macrophages showed that the nanoplatforms decreased the immunostimulatory potential and avoided the activation of the immune system. Li et al. made use of macrophage-derived microvesicles by stimulating cytochalasin B to coat PLGA nanoparticles loaded with tacrolimus (T-MNPs) (Fig. 8A) [139]. The in vitro binding of T-MNPs to inflamed HUVECs was significantly stronger than that of erythrocyte membrane-coated nanoparticles (RNPs). The results showed that macrophage membrane-coated nanoparticles significantly enhanced in vivo targeting in mice with collagen-induced arthritis compared with DIR, bare NPs and RNP. The in vivo tissue distribution further clarified the better targeting capacity of the MNPs to the paws than the others. Proteomic analysis has revealed the targeting mechanism, suggesting that MAC-1 and CD44 contribute to the prominent targeting of MNPs. Therefore, in vivo immunofluorescence revealed higher levels of P-selectin and ICAM-1 expression in the arthritic paw (Fig. 8B). The inflamed endothelium in RA highly expresses P-selectin and ICAM-1, which can be recognized by CD44 and MAC-1. The in vivo drug release of T-MNPs demonstrated sustained release and long retention. The expression of proinflammatory cytokines provided that T-MNPs could remarkably reduce their production. The therapeutic effect of RA showed that T-MNPs significantly suppressed RA and maintained a stable period (Fig. 8C). H&E staining of joint tissues further confirmed this effect.

More recently, Li’s team reported that macrophage-derived microvesicle membranes coated nanoparticles to treat RA [65, 137]. One used M2-type exosomes to load plasmids encoding IL-10 cytokine and betamethasone sodium phosphate, and another used exosome membranes to coat zeolitic imidazolate framework-8 (ZIF-8)-loaded dexamethasone sodium phosphate. All studies have indicated that the macrophage biomimetic platform could be applied to RA and is a promising potent for other immune diseases.

### Other diseases

Macrophages are the cells of the immune system. They can phagocytose foreign materials from bacteria and debris to dead cells. A large number of leukocytes migrate to the inflamed lesion to regulate inflammatory processes. Macrophage membranes and macrophage-derived microvesicles are used to deliver the drug to the lesions.

Tang et al*.* generated dexamethasone-loaded macrophage-derived microvesicles (MV-DXs) for the treatment of renal inflammation and renal fibrosis [140]. The high expression of integrin α_L_β_2_ (LFA-1) and α_4_β_1_ (VAL-4) caused them to adhere to the inflamed kidney. Furthermore, MV-DEX significantly decreased renal injury in murine models of LPS- or ADR-induced nephropathy compared with free DEX. Meanwhile, MV-DEX sharply reduced the side effect of DEX due to the precise targetability. Ulcerative colitis (UC) is characterized by relapsing inflammation in the colon. Sun et al*.* developed a macrophage membrane-coated ROS-sensitive β-cyclodextrin loaded with rosiglitazone (RMN NPs) to treat UC [39]. Owing to the inflammatory homing effects, the macrophage membrane assisted RMN NPs in inflammatory colonic tissues to suppress inflammation. Based on the overproduction of ROS and upregulation of transmembrane glycoprotein CD98 in UC, Ma et al. developed a macrophage membrane to coat a pH-responsive MOF carrier loaded with carbon nanodots and CD98 CRISPR/Cas9 plasmid (CCZM) [141]. The results of cellular uptake in RAW264.7 cells showed that macrophage membrane-coated nanoparticles had a 2.5-fold higher fluorescence intensity than nanoparticles without membrane decoration. Regarding the in vivo inflammation targeting ability of the macrophage membrane, CCZM demonstrated significantly stronger fluorescence in the inflammatory colon. The combined effect of nanozymes and the CRISPR/Cas9 system significantly alleviated UC inflammation and provided a promising approach for the personalized treatment of inflammation. Zhang et al*.* reported a biomimetic nanoparticle with a ‘lure and kill’ mechanism that was made by embedding with a macrophage membrane doped with a PLGA core loaded with melittin and MJ-33 for pancreatitis (MФ-NP) [142]. In pancreatitis models, MФ-NP effectively inhibited PLA2 activity and PLA2-induced pancreatic injury. In allergic asthma (AA), the adoptive transfer of M2 macrophages could aggravate pathologies of allergic airway inflammation. Pei et al. explored the exosome membrane of M2 macrophages coated PLGA@Dnmt3aos^smart silencer^ to treat AA [143]. Dnmt3aos^smart silencer^ is a small interfering RNA (siRNA) that is easily degraded. Therefore, they established PLGA NPs to obtain a nanocomplex with siRNA and exosome decoration to improve the biodistribution, stability, efficacy and biocompatibility of NPs. The encapsulation efficiency of Dnmt3aos^smart silencer^ into PLGA was determined to be 70.70 ± 1.82%. The release kinetics of the Dnmt3aos^smart silencer^ showed a sustained release profile of more than 50% of the cumulative release rate at 24 h. Teo et al*.* designed macrophage membrane-coated NPs originating from M0, M1 and M2 macrophage membranes for the treatment of osteoarthritis [144]. Interestingly, M2 macrophage membrane-coated NPs showed superior efficacy in sponging proinflammatory cytokines and relieving osteoarthritis.

In conclusion, macrophage membrane-mimetic nanoparticles can effectively target inflamed tissue and mitigate the inflammatory response by utilizing the natural inflammatory targeting effect of macrophage membranes. Meanwhile, the anti-inflammatory type of M2 macrophages exhibited a superior anti-inflammatory effect. This lays the foundation for the clinical application of cell membrane-mimetic nanoparticles loaded with clinical applications.

## Relevance to clinical studies

Over the past years, the development and application of macrophage-coated nanoparticles have been an increasing number of relevant patents published. As shown in Table 4, these patents of drug delivery were initially performed by using macrophages to directly carry cargoes. Subsequently, macrophage membranes were used to wrap various nanomaterials with more diversified therapeutic actions. Then, modified macrophage membranes were used as the envelope material. These patents show that macrophage membrane bionic nanoparticles can combine with other therapy, offering a potential novel platform for disease treatment. And the mimetic platform was progressing in a more efficient and intelligent direction in the past years.

**Table 4:** Patents of macrophage membrane coating technology

Patent number	Patent title	Assignee	Filing year
CN201711173732.0	Preparation and application of biomimetic drug delivery system of cell membrane targeting atherosclerotic lesions	Southwest University, Chongqing, China	2017
CN201811572702.1	Macrophage vesicle entrapped nano-drug preparation and application thereof in treating arthritis	Fudan University, Shanghai, China	2018
CN201811572702.1	Macrophage vesicle entrapped nano-drug preparation and application thereof in treating arthritis	Fudan University, Shanghai, China	2018
CN201811022034.5	Preparation and application of cytomembrane biomimetic lipoprotein targeted nanometer drug delivery system	China Pharmaceutical University, Jiangsu, China	2018
CN201910888938.4	Preparation method for lysosomal membrane coated nanoparticle	Beijing University of Chemical Technology, Beijing, China	2019
CN201911242023.2	Preparation method and application of macrophage membrane bionic bismuth selenide nanoparticles	Zhengzhou University, Henan, China	2019
CN201910806494.5	Anti-breast cancer nano-drug and preparation method thereof	Suxin, Jilin, China	2019
CN201910964752.2	Bionic nano-drug for preventing and treating aortic dissection and preparation method thereof	Fudan University, Shanghai, China	2019
CN201910610087.7	Oxygen self-supply type targeted nano photodynamic therapy system	Shanghai Jiaotong University School of Medicine, Shanghai, China	2019
CN202010033464.8	Targeted delivery system based on functionalized macrophages/monocytes, and construction and application of targeted delivery system	Xidian University,	2020
		Shanxi, China	
CN202010405924.5	Preparation method of double-membrane-coated bionic nano diagnosis and treatment probe	Nankai University, Tianjin, China	2020
CN202010510913.3	Multi-targeting fusion cell membrane modified bionic nano delivery system as well as preparation method and application thereof	Shanghai Ninth People's Hospital, Shanghai Jiao Tong University School of Medicine, Shanghai, China	2020
CN202010555452.1	Bionic nano material for sonodynamic/gas synergistic anti-tumor treatment and preparation method of bionic nano material	Mengchao Hepatobiliary Hospital of Fujian Medical University, Fujian, China	2020
CN202011226922.6	Cantharidin-loaded macrophage membrane encapsulated metal organic framework nanoparticle and preparation method thereof	Dalian University of Technology, Liaoning, China	2020
CN202011212257.5	Alveolar macrophage-like multifunctional nanoparticle loaded with aggregation-induced emission photothermal material and preparation method and use of alveolar macrophage-like multifunctional nanoparticle	The Fifth Affiliated Hospital, sun yat-sen University, Guangdong, China	2020
CN202011638846.X	Bionic nano-drug targeting myocardial infarction locality and preparation method thereof	Shanghai Chest Hospital, Shanghai, China	2020
CN202010975805.3	Synthesis method of bionic macrophage membrane nano drug-loaded particles and application of bionic macrophage membrane nano drug-loaded particles in new coronavirus pneumonia	Affiliated Fifth Hospital of Zhongshan University, Guangdong, China	2020
CN202110800585.5	Bionic nano hydrogel coated with a macrophage membrane and loaded with manganese dioxide MnO2 and cis-platinum Pt as well as preparation and application thereof	Donghua University, Shanghai, China	2021
CN202110628432.7	Bionic nano-drug co-loaded with JTC801 and DNA methylated transferase inhibitor and preparation method and application thereof	Zhengzhou University, Henan, China	2021
CN202110478192.7	Choroidal neovascularization targeting nanoparticle coated with macrophage membrane and preparation method of choroidal neovascularization targeting nanoparticle	Chen Zhao, Shanghai, China	2021
CN202110969245.5	Gene editing prodrug system for treating colitis and application of gene editing prodrug system	Zhejiang University, Zhejiang, China	2021
CN202111171093.0	Nano-drug for inhibiting injured vascular intimal hyperplasia and application of nano-drug	Chongqing University, Chongqing, China	2021
CN202111065311.2	Macrophage membrane coated arginine deiminase/catalase/IR780 nanoparticle, preparation method and application	Chongqing Medical University, Chongqing, China	2021
CN202111275670.0	Bionic nano bait, and preparation method thereof and application of bionic nano bait in sepsis treatment	Shandong University; Suzhou Research Institute, Shandong University, Shandong, China	2021
CN202111491404.1	-	Hunan University of Chinese Medicine, Hunan, China	2021
CN202111286130.2	-	Air Force Medical University, Shanxi, China	2021
CN202110117736.7	-	Academy of Military Sciences, Beijing, China	2021
CN202210044973.X	​Gold/manganese bionic nano material as well as preparation method and application thereof in preparation of tumor diagnosis and treatment drugs	Stomatological Hospital of Southern Medical University, Guangdong, China	2022
CN202210564556.8	-	Zhejiang University, Zhejiang, China	2022
CN202210575019.3	-	Union Hospital Tongji Medical college Huazhong University of Science and Technology, Hubei, China	2022
CN202210038633.6	​Cell membrane coated nano bait for removing pro-inflammatory factors and inhibiting T cell activation and preparation method and application thereof	Suzhou University, Jiangsu, China	2022
CN202210104890.5	Nanometer delivery system capable of promoting permeation, relieving tumor hypoxia and targeting tumor cells as well as preparation method and application of nanometer delivery system	First Auxiliary Hospital of Chinese People Liberation Navy Military University, Shanghai, China	2022
CN202210038633.6	-	Suzhou University, Jiangsu, China	2022

## Summary and future challenges

Cell membrane cloaking is a potential platform for drug delivery and therapy strategies. As a type of immune monocyte cell, macrophages are key factors in the physiological functions and pathology of various diseases. Macrophage membrane-coated nanoparticles enhance the retention of the loaded drug. Moreover, macrophage membrane-coated nanoparticles can target inflamed sites or tumor sites and neutralize endotoxins, thus achieving safe and efficient therapeutic effects. This paper reviews the preparation, characterization, application and clinical challenges of macrophage membrane-coated core nanoparticles. The mimetic strategy has been studied in the treatment of cancer, immune disease, atherosclerosis, infection, and inflammatory disease. The above discussion also proves that macrophages are potential cell membrane donors. It is expected that macrophage membrane cloaking will be translated into clinical trials and act as an important part in the field of biomedicine.

However, there are many challenges faced in clinical translation. Firstly, how to obtain macrophage membranes on a large scale under the premise of ensuring the biological function of membrane proteins. Secondly, among the currently known coating methods, including ultrasound, coextrusion and electroporation, the membrane can be successfully coated on the surface of nanoparticles, but it is difficult to ensure the coating efficiency in large-scale processing. To ensure consistent physical and chemical parameters, the preparation and processing methods of different nanoparticles are also different. Therefore, it is necessary to further optimize membrane-coated nanoparticle technology and develop a simple, efficient and large-scale preparation of new technology. Moreover, ultralong-term stability studies should be conducted under different storage conditions to ensure the efficacy and stability of the product. To our knowledge, macrophage membrane-encapsulated nanoparticles can be used in a variety of diseases to enhance therapeutic efficacy in vivo and in vitro. However, the biosafety of cell membranes should be carefully studied before applying in clinical trials. Firstly, immunogenicity between immune cell membrane donors and acceptors may lead to serious safety concerns. Secondly, certain specific modifications of cell membranes may also lead to some unexpected side effects. Thirdly, issues about pharmacokinetics, safety, and interaction with materials in the body should be addressed to improve the clinical translation of macrophage membrane cloaking. More importantly, there is no formal regulatory standard for bionic products, so more attention should be given to the safety and effectiveness of bionic nanoparticles in humans. Therefore, in future studies, these limitations must be overcome before such methods can be used as standards for clinical treatment.

## Data Availability

Not applicable.

## Abbreviations

FDA: Food and drug administration
PLGA: Poly [D, L-lactic-co-glycolic acid]
PLA: Polylactic acid
PCL: Polycaprolactone
EPR: Enhanced permeability and retention
PEG: Polyethylene glycol
ABC: Accelerated blood clearance
RES: Reticuloendothelial system
PRRs: Pattern recognition receptors
DAMPs: Damage associated molecular patterns
PAMPS: Pathogen-associated molecular patterns
LPS: Lipopolysaccharide
IFN-γ: Interferon-γ
TNF-α: Transforming factor-α
IL-4: Interleukin-4
IL-10: Interleukin-10
IL-13: Interleukin-13
IL-21: Interleukin-21
ECM: Cell-extracellular matrix
PSGL-1: P-selectin glycoprotein ligand-1
LFA-1: Lymphocyte function-associated antigen 1
VLA-4: Very late antigen-4
VCAM-1: Vascular cell adhesion protein 1
UCNPs: Upconversion nanoparticles
MOFs: Metal-organic frameworks
DLS: Dynamic light scattering
TEM: Transmission electron microscopy
SEM: Scanning electron microscopy
SDS-PAGE: Sodium dodecyl polyacrylamide gel electrophoresis
PTX: Paclitaxel
PTT: Photothermal therapy
PDT: Photodynamic therapy
BMMs: Bone marrow derived macrophages
DDS: Drug delivery system
TAMMs: Tumor associated macrophage membranes
CSS: Cytokine storm syndrome
ACE II: Angiotensin converting enzyme 2
RA: Rheumatoid arthritis
AD: Alzheimer’s disease
PD: Parkinson’s disease
ALS: Amyotrophic lateral sclerosis
BBB: Blood-brain barrier
SCI: Spinal cord injury
NGF: Nerve growth factor
AS: Atherosclerosis
